# Baicalin Ameliorates Pancreatic Fibrosis by Inhibiting the Activation of Pancreatic Stellate Cells in Mice with Chronic Pancreatitis

**DOI:** 10.3389/fphar.2020.607133

**Published:** 2021-01-18

**Authors:** Jianwei Fan, Lifang Duan, Nan Wu, Xiaofan Xu, Jiaqi Xin, Shengnan Jiang, Cheng Zhang, Hong Zhang

**Affiliations:** ^1^ Basic Medical Academy, Shaanxi University of Chinese Medicine, Xianyang, China; ^2^ Medical Experiment Center, Shaanxi University of Chinese Medicine, Xianyang, China; ^3^ Department of Hepatobiliary Surgery, Xianyang Central Hospital, Xianyang, China

**Keywords:** baicalin, pancreatic inflammation, pancreatic fibrosis, pancreatic stellate cells, nuclear factor-κB

## Abstract

Pancreatic inflammation and fibrosis are typical pathological features in chronic pancreatitis (CP). Activated pancreatic stellate cells (PSCs) have been regarded as the core event in the development of pancreatic fibrosis and are considered to be the key target for treatment of CP. Baicalin (C_21_H_18_O_11_), the main chemical composition of Baikal skullcap in the traditional Chinese medicines Dachaihu decoction (DCHD) and Xiaochaihu decoction (XCHD), has shown significant effects in the treatment of pancreatic fibrosis in CP mice; however, whether baicalin can inhibit the activation of PSCs and its underlying mechanism remain unclear. In this study, the influence of baicalin on activated PSCs *in vitro* and *in vivo* was investigated, and the results showed that Baicalin could significantly ameliorate the degree of pancreatic inflammation and fibrosis, while decreasing the levels of alpha-smooth muscle actin (α-SMA), F4/80 (surface markers of mouse macrophages), nuclear factor kappa-B (NF-κB), monocyte chemotactic protein 1 (MCP-1), and collagen type I alpha 1 (COL1A1)in the pancreas. Moreover, NF-κB and α-SMA were co-expressed in the pancreas of CP mice. Baicalin treatment markedly reduced the expression of co-location of α-SMA and NF-κB. *In vitro*, the protein expression levels of transforming growth factor-β receptor 1 (TGF-βR1), phosphorylated TGF-β activated kinase 1 p-TAK 1, and NF-κBp65 in PSCs were all remarkably reduced after treatment with baicalin. In addition, baicalin could inhibit MCP-1 mRNA expression in supernatant of activated PSCs, as well as the excessive migration of macrophages. Taken together, our findings indicated that baicalin could inhibit the TGF-β1/TGF-βR1/TAK1/NF-κB signaling pathway of activated PSCs, reduce the secretion of MCP-1, and further decrease the infiltration of macrophages and inflammation cells of the local microenvironment of the pancreas. Thus, this study provides a reliable experimental basis for baicalin in the prevention and treatment of CP.

## Introduction

Chronic pancreatitis (CP) is characterized by progressive and irreversible injury in the pancreas caused by various etiologic factors, ultimately leading to endocrine and exocrine dysfunction (Chari 2016). Clinical manifestations include persistent or recurrent abdominal pain, anorexia and steatorrhea, and even lead to diabetes mellitus, which seriously affects the quality of life of patients (Lew et al., 2017). CP has been considered as the important risk of pancreatic cancer and showed a nearly 50% mortality rate within 20–25 years of diagnosis (Lankisch et al., 1993). Pancreatic fibrosis has been considered not only a common pathological feature but also the core event in the progression of CP, as well as the key target for treatment (Conti et al., 2018; Xu et al., 2018).

Up to now, the CP treatment has concentrated on some supportive therapies for helping patients to alleviate clinical symptoms, correct complications, and improve life quality. As for pancreatic fibrosis, there have been no effective suppressing measures in clinical application. Recently, some Chinese herbal medicines have been used in the treatment of CP (Xiao, 2019; Shi et al., 2020). It was reported that classical Chinese prescriptions, such as Dachaihu decoction (DCHD) and Xiaochaihu decoction (XCHD), have shown significant clinical benefits for CP patients (Liu C. et al., 2019; Cui et al., 2017; Shi et al., 2020), such as improving the digestive manifestation of patients. Moreover, our previous experiments confirmed that DCHD could remarkably reduce the degree of pancreatic fibrosis in L-arginine-induced CP mice (Xu et al., 2016; Duan et al., 2017).

Baikal skullcap is an important ingredient of DCHD and XCHD, which has been used for removing heat and toxic materials, stanching bleeding and so on (Zhu et al., 2020). Baicalin (C_21_H_18_O_11_) is the main chemical composition of Baikal skullcap, it is a kind of flavonoid extracted from the dried root of Baikal skullcap and has a wide range of pharmacological effects, such as anti-inflammation, scavenging oxygen free radicals, and anti fibrosis in many organs including liver, kidneys and lung (Wang et al., 2015; Dinda et al., 2017; Chen et al., 2019; Zhao et al., 2020). However, it remains unclear whether Baicalin can inhibit pancreatic inflammation and fibrosis in CP, and its exact mechanism is worthy of further study.

Many experiments have demonstrated that the activation of pancreatic stellate cells (PSCs) and subsequent production of extracellular matrix (ECM) play critical roles in the progression of pancreatic fibrosis in CP (Bynigeri et al., 2017; Li et al., 2018). PSCs remains quiescent under physiological conditions, but they are activated once the pancreas is exposed to various damage factors, especially the inflammatory factors released by the local infiltrated inflammatory cells in the microenvironment of the pancreas (Sun et al., 2018; Xue et al., 2018). However, whether baicalin interferes with the activation of PSCs, as well as its detailed molecular mechanism, remains unknown. Therefore, in this research, we aimed to study the effect of baicalin on PSC activation *in vitro* and *in vivo* to further elucidate the concrete mechanism of baicalin inhibiting pancreatic fibrosis.

## Materials and Methods

### Animals

C57BL/6 male mice (male, 6 weeks old, 18–25 g), obtained from the animal experiment center of Medical College of Xi’an Jiaotong University [Certificate SCXK (Shan) 2018-001], were housed under a controlled environment (22° ± 2°C); tap water and standard laboratory diet were freely provided. Before the experiment, all mice were raised adaptively for 1 week. The mice received humane care in accordance with “The Guide for Care and Use of Laboratory Animals” issued by Shaanxi University of Traditional Chinese Medicine.

### Experimental Model of Chronic Pancreatitis

The mice were randomly divided into the three following groups (per time point in each group, n = 8): a control group, CP group, and baicalin group. Mice in the CP and baicalin groups were injected intraperitoneally with cerulein (C9026; Sigma Aldrich, Darmstadt, Germany) to induce CP (50 μg/kg, 6 times per day, 3 days/week for 6 weeks), and the same volume of sterilized physiological saline was injected into the control mice.

**FIGURE 1:**
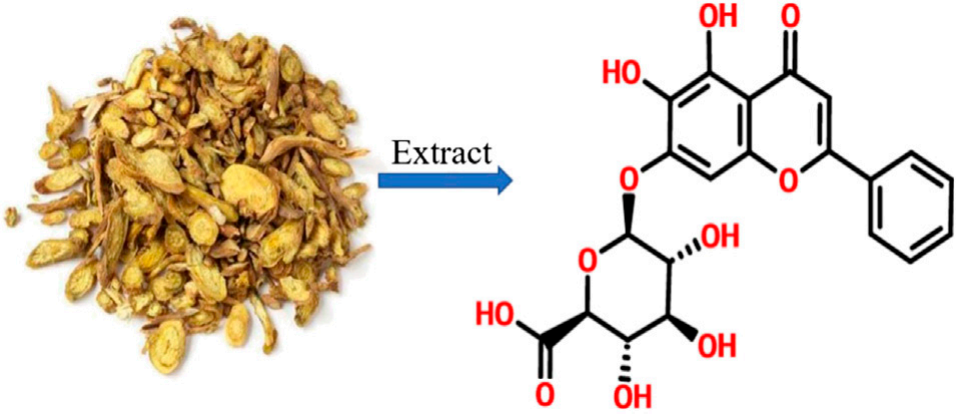
Chemical structure of baicalin.

Baicalin (chemical structure as shown in Figure 1) (21967-41-9, Shanghai Dibai Chemical Technology Co., shanghai, China) with a purity of 98%. The preparation of baicalin solution *in vivo*: the dry powder of baicalin monomer was dissolved with double distilled water to make up 10 mg/ml of baicalin solution, and injected intraperitoneally (100 mg/kg/day, 6 days/week) into the mice of the baicalin group 2 weeks after the first administration of cerulein. *In vitro*, baicalin was dissolved with high glucose DMEM to make up to 50 μg/ml.

At 2, 4, and 6 weeks after modeling, the mice were anesthetized and sacrificed, and the pancreas was completely removed, immediately immersed in 10% neutral formaldehyde solution for fixation or quickly put into liquid nitrogen, and then transferred to a −80°C refrigerator for subsequent extraction of RNA and protein. Blood from the inferior vena cava was drawn for detection of the serum index.

### Histology and Masson Staining

After dehydration, paraffin embedding, and sectioning (3 μm) of the pancreas, hematoxylin and eosin and Masson staining protocols were carried out according to the method in the manual to observe the morphological and fibrosis changes in the pancreas.

### Immunohistochemistry and Immunofluorescence Staining

Immunohistochemistry (IHC) was performed to evaluate the expressions of alpha-smooth muscle actin (α-SMA), F4/80 (surface markers of mouse macrophages), nuclear factor kappa-B (NF-κB), monocyte chemotactic protein 1 (MCP-1), and collagen type I alpha 1 (COL1A1). The sections (3 μm) were dewaxed with water, boiled with citric acid buffer for antigen repair, and blocked with 3% H_2_O_2_ for 10 min and 5% bovine serum albumin (BSA) for 40 min. Antibodies were applied overnight at 4°C at the following concentrations: anti-F4/80 antibody (sc-71088, Santa Cruz, 1:200), anti-MCP-1 antibody (bs-1101R, Bioss, 1:200), anti-NF-κB p65 antibody (sc-8008, Santa Cruz, 1:200), anti-α-SMA antibody (bs-10196R, Bioss, 1:200), and anti-COL1A1 antibody (sc-25974, Santa Cruz, 1:200). They were then incubated with the instant biotinylated secondary antibody for 1 h according to the standard method, followed by visualization, counterstaining with hematoxylin, dehydration, and mounting.

Dual immunofluorescence staining was performed to detect the co-expression of α-SMA and NF-κB p65, followed by incubation overnight at 4°C with anti-α-SMA antibody (bs-10196R, Bioss, 1:250) and anti-NF-κB p65 antibody (sc-8008, Santa Cruz, 1:150). The samples were incubated with fluorescein isothiocyanate (FITC)-labeled goat antirabbit secondary antibody and CY3-labeled goat anti mouse IgG (1:400 dilution) for 1.5 h. 4′,6-diamidino-2-phenylindole (DAPI) (1:1,000) was used to counterstain the nuclei for 5–15 s; the coverslips were covered with anti-fade mounting medium, and images were collected using laser scanning confocal microscopy (FV3000, Olympus, Tokyo, Japan).

### Isolation of Mouse Bone Marrow–Derived Macrophages

To isolate mouse bone marrow–derived macrophages (BMDMs), we first rinsed the bone marrow cells of the femur with serum-free 1,640 solution and cultured them in a complete medium containing 10% fetal bovine serum for 16 h. We then collected the supernatant and centrifuged it. After the cells were resuspended in complete medium, M-CSF (50 ng/ml) was added and inoculated on a 12-well culture plate. Five days later, mature macrophages were harvested, the complete culture medium was replaced with serum-free 1,640 medium and left for another 12 h, and the wound healing assay was carried out to observe the migration ability of macrophages to the damaged site.

### Isolation and Culture of Mouse Pancreatic Stellate Cells

Following the method we used in our previous study (Apte et al., 1998; Fan et al., 2012), we isolated the PSCs of healthy C57BL/6 male mice, cultured them with Dulbecco’s Modified Eagle’s Medium (DMEM) containing 15% fetal bovine serum to the second generation, and inoculated them on porous cell culture plates. After 4 h, the culture medium was completely changed with serum-free DMEM culture medium. Cells were synchronized in an incubator for 24 h at conditions of 37°C and 5% CO_2_. The PSCs were divided into three groups, as follows: a control group, a transforming growth factor-β (TGF-β) group (5 ng/ml), and a TGF-β1 (5 ng/ml) + baicalin (50 ug/ml) group. The culture supernatant and cells were collected at the corresponding time. NF-κB and α-SMA protein in PSCs were extracted and detected by Western blot. The MCP-1 level in the supernatant was detected by enzyme-linked immunosorbent assay (ELISA), and the effect of PSC supernatant on the migration ability of macrophages was observed.

### Total RNA Extraction and Real-Time Polymerase Chain Reaction

Total tissue RNA was extracted using an RNA Extraction Kit (9767, Takara Bio, Dalian, China) according to the manufacturer’s instructions. Total RNA was reversed transcribed into cDNA using reverse transcriptase kits (RR036A; Takara Bio, Dalian, China) and OligodT primers according to the manufacturer’s guidelines. Using cDNA as a template for PCR, the whole real-time polymerase chain reaction (PCR) reaction was performed in a 20 μL mixture, including 10 μL of SYBR Premix DimerEraser, 0.8 μL of upstream and downstream primer (10 μmol/L) respectively, 0.08 μL of Rox reference dye II, 7 μL of cDNA (20 ng/μL), and 1.32 μL of ddH2O. Real-time quantitation was performed using an ABI-7500 Sequence Detection System (Thermo Fisher Scientific, Shanghai, China). The PCR conditions were as follows: cycle preheating for 2 min at 50°C, cycle pre-denaturation for 10 min at 95°C, denaturation for 5 s at 95°C, annealing for 30 s at 60°C, and extension for 30 s at 72°C, with 40 cycles in total. Glyceraldehyde-3-phosphate dehydrogenase (GAPDH) was used as the internal control to calculate the relative expression of the target gene. PCR primers are shown in Table 1.

**TABLE 1:** The sequences of the primers for real-time PCR.

Gene	The primer sequence (5′-3′)	Cycles	Tm (°C)
MCP-1	Forward: GTT​GGC​TCA​GCC​AGA​TGC​A	40	60
	Reverse: AGCGTACTCATTGGGATVATCTTG		
GAPDH	Forward: TGA​ACG​GGA​AGC​TCA​CTG​G	40	60
	Reverse: TCC​ACC​ACC​CTG​TTG​CTG​TA		

### Protein Extraction and Western Blotting

Cellular proteins were prepared from mouse pancreas and PSCs using standard methods. Protein concentrations were measured using a BCA protein assay kit (Boster); the samples were adjusted to 4 μg/μL using 6 × loading buffer (TransGen Biotech, Beijing, China) and boiled at 95°C for 5 min. Protein extracts were subjected to sodium dodecyl sulfate–polyacrylamide gel electrophoresis (SDS-PAGE; Bio-Rad, Hercules, CA, United States) and blotted to polyvinylidene difluoride membrane (EMD Millipore, Darmstadt, Germany). The membranes were blocked with 5% non-fat milk for 1 h, then probed with the following primary antibodies: anti-GAPDH (BM1623, Boster, 1:1,000), anti-α-SMA (bs-10196R, Bioss, 1:400), anti-NF-κB p65 (sc-8008, Santa Cruz, 1:400), anti-p-NF-κB p65 (#3033, CST, 1:1,000), anti-COL1A1 (#84366, CST, 1:1,000), TGF-βR1 (SC-402, Santa Cruz, 1:400), and p-TAK1 (bs-5435R, Bioss, 1:400), respectively, overnight at 4°C. The samples were then incubated with horseradish peroxidase conjugated second antibody (goat anti-rabbit, goat anti-mouse, or rabbit anti-goat, 1:2,000) for 1 h. A 10–250 kDa protein marker was used to determine the size of detected bands in the BeyoECL Star Chemiluminescent system (Beyotime Biotechnology, Shanghai, China).

### Measurement of Cytokine Levels

The level of MCP-1 in the supernatant of PSCs was detected using an ELISA Kit (ek0411, Boster) according to the standard method provided by the manufacturer.

### Detection of Macrophage Migration Using the Wound Healing Assay

The BMDMs were inoculated into 12-well culture plates with serum-free 1,640 medium at 1 × 10^6^. Twelve hours later, a straight scratch was made on the BMDMs using the tip of a P200 pipette. The cells were washed with phosphate-buffered saline (PBS) three times and incubated with different sources of PSC supernatant, including the control group, the TGF-β1 group, and the TGF-β1 + baicalin group. After incubation for 0 and 24 h, the gap width of scratch re-population was measured and compared with that of 0 h.

### Statistical Analysis

Data are presented as the mean ± standard deviation (SD). Differences were analyzed using a *t*-test and one-way analysis of variance. A *p*-value ≤0.05 was considered statistically significant.

## Results

### Baicalin Alleviated Pancreatic Injury and Fibrosis in Chronic Pancreatitis Mice

**FIGURE 2:**
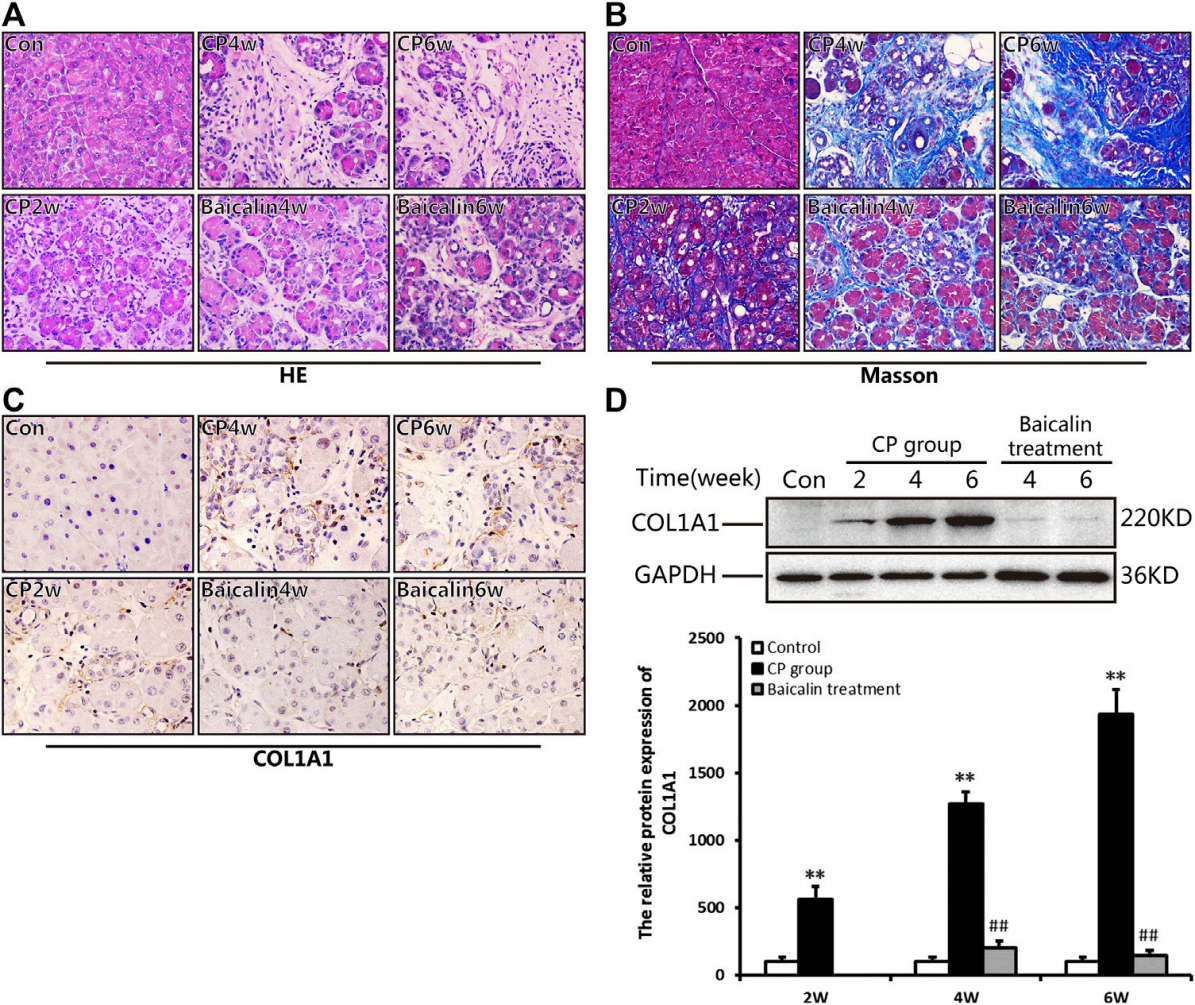
Baicalin protects against pancreatic injury and pancreatic fibrosis in mice with chronic pancreatitis (CP) induced by cerulein. **(A)** Changes in pancreatic morphology at different timepoints were evaluated by hematoxylin and eosin staining (original magnification, ×200). **(B)** Collagen deposition was detected by Masson staining (original magnification, ×200). **(C)** Immunohistochemistry of COL1A1was performed to evaluate the degree of pancreatic fibrosis, and the brown color shows positively stained cells. (original magnification, ×400). **(D)** Protein expression of COL1A1 in pancreas was analyzed by Western blot, and GAPDH was used as an internal control; the relative protein abundance was quantified by densitometry analysis. Values are shown as mean ± SD (*n* = 3). **p < 0.01 vs. control; ^##^ *p* < 0.01 vs CP group.

As shown in Figure 2A, 2 weeks after modeling, the pancreas exhibited obvious edema, inflammatory cell infiltration, acinar atrophy with a small amount of necrosis, and slight deposition of collagen fibers. At 4 weeks, more obvious pancreatic injury was observed, including tubular complexes, more inflammatory cells and marked fibrosis. At 6 weeks, the structure of the pancreatic lobules was severely damaged, and many acinar cells were replaced by tubular complexes or interconnected collagen deposition. Interestingly, after administration with baicalin, the degree of pancreatic injury, both inflammatory cells and pancreatic fibrosis, were all significantly alleviated, showing only a few instances of acinar atrophy, tubular complexes, and collagen deposition compared with that in the CP group at the same timepoint.

Masson staining was performed to evaluate the degree of pancreatic fibrosis. The results showed a small amount of collagen deposition in the pancreas at 2 weeks after modeling, which was increased significantly at 4 and 6 weeks (Figure 2B). However, baicalin treatment remarkably reduced the amount of collagen deposition in the pancreas.

To further confirm the above result, we performed IHC and Western blot to detect the positive staining ratio and protein expression level of COL1A1, which is an indicator of fibrosis. The results showed that more positive staining ratio and higher expression of COL1A1 were observed in the pancreas with the development of CP (*p* < 0.01), but the level of COL1A1 expression were significantly downregulated after treatment with baicalin (*p* < 0.01) compared with that in the CP group at the same timepoint (Figures 2C,D). Overall, these findings provide evidence that baicalin is available to alleviate pancreatic injury and fibrosis in CP mice.

### Baicalin Inhibited Pancreatic Stellate Cells Activation in the Development of Chronic Pancreatitis

**FIGURE 3:**
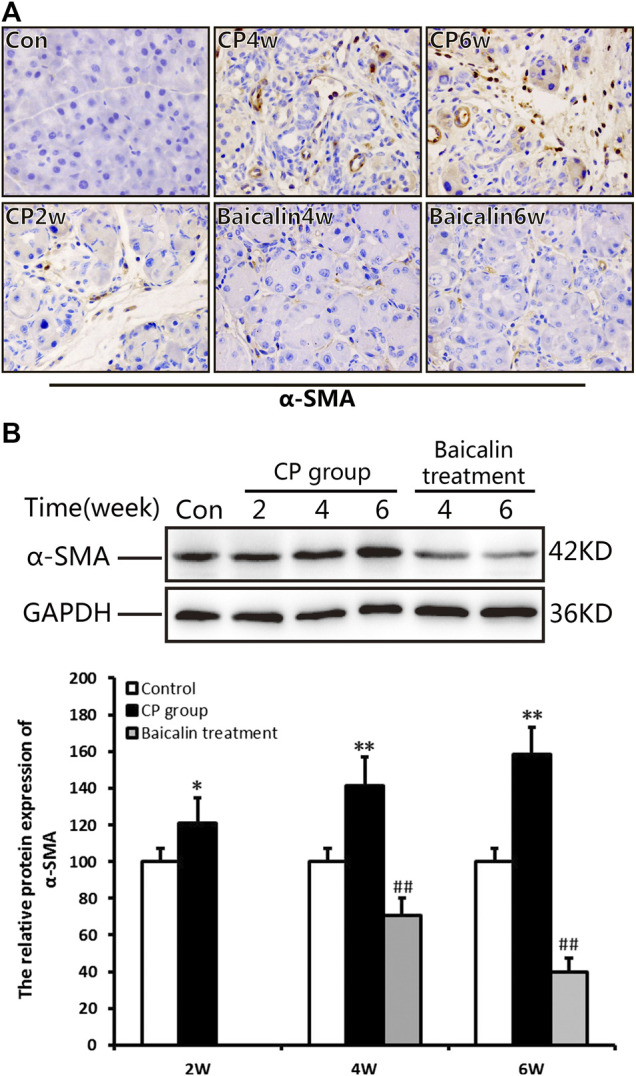
Effect of baicalin on PSC activation. **(A)** Immunohistochemistry of α-SMA associated with CP. The brown color shows positive cells (original magnification, ×400). **(B)** The protein expression of α-SMA in pancreas was analyzed using a Western blot, and GAPDH was used as an internal control; relative protein abundance was quantified by densitometry analysis. Values are shown as mean ± SD (*n* = 3). **p < 0.01 vs. control; ^##^ *p* < 0.01 vs. CP group.

As shown in Figure 3A, there was no α-SMA positive staining in the normal pancreas except for the pancreatic duct structure. Cerulein resulted in obvious positive staining of α-SMA in cellular plasma of PSCs at 4 and 6 weeks after modeling. The results of Western blot further confirmed the overexpression of α-SMA in the pancreas after CP. However, baicalin treatment could significantly reduce the intensity of positive staining and protein level of α-SMA at 4 and 6 weeks, suggesting that baicalin can inhibit the activation of PSC in the pancreas (Figures 3A,B).

### Baicalin Inhibited Nuclear Factor Kappa-B (NF-κB) Expression in the Pancreas and Pancreatic Stellate Cells

**FIGURE 4:**
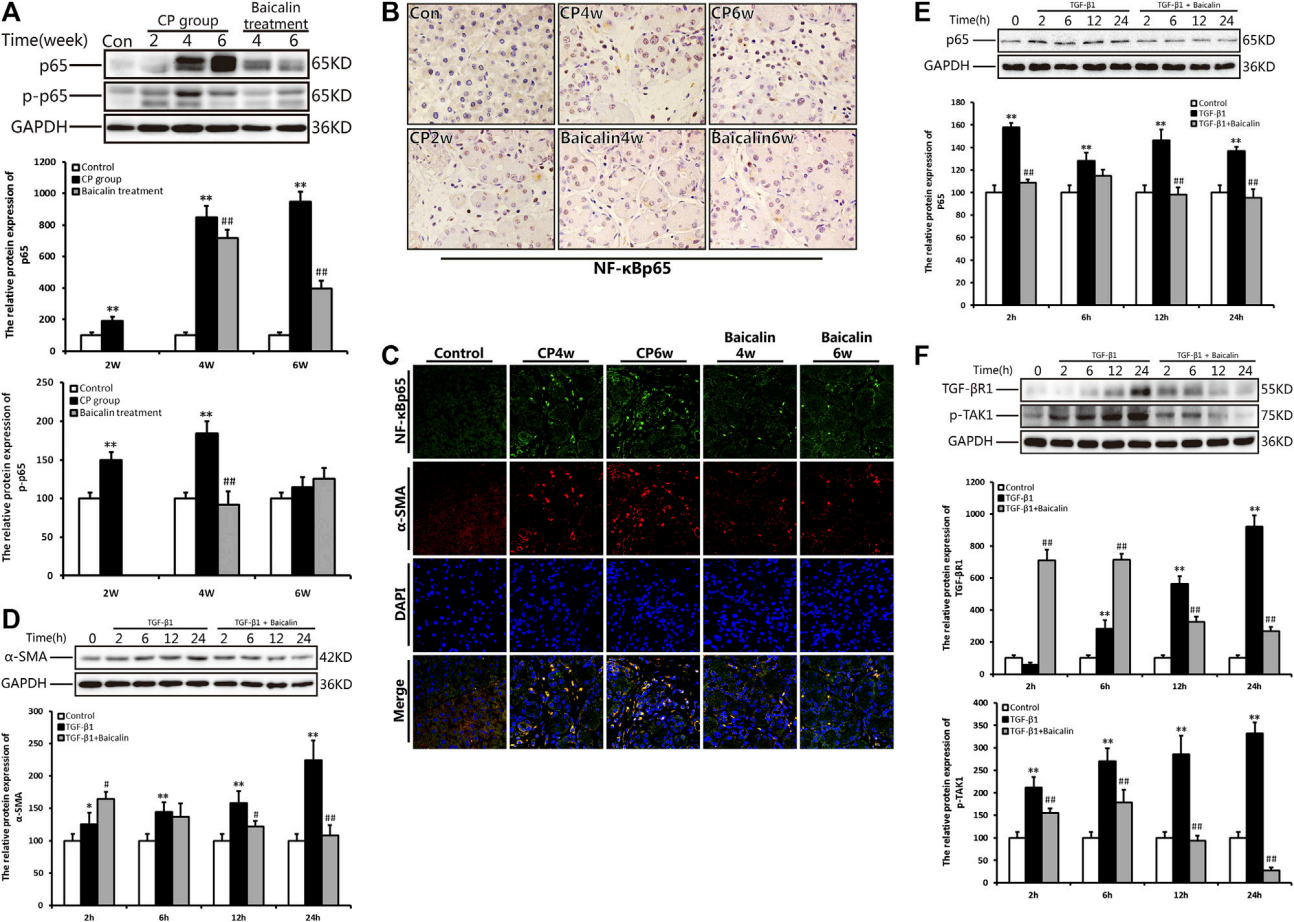
Effect of baicalin on NF-κB expression in the pancreas and PSCs. **(A)** The protein expressions of NF-κBp65 and p-p65 (phosphorylated p65) in the pancreas was analyzed using a Western blot, and GAPDH was used as an internal control; relative protein abundance was quantified by densitometry analysis. Values are shown as mean ± SD (*n* = 3). **p < 0.01 vs. control; ^##^ *p* < 0.01 vs. CP group. **(B)** Immunohistochemistry of NF-κBp65 associated with CP. The brown color shows positive cells (original magnification, × 400). **(C)** Double immunofluorescent signals of NF-κB and α-SMA, showing co-expression in different groups (original magnification, ×400). **(D) **The protein expression of α-SMA in PSCs in different groups was analyzed using Western blot, and GAPDH was used as an internal control. Relative protein abundance was quantified by densitometry analysis. Values are shown as mean ± SD (*n* = 3). *p < 0.05, **p < 0.01 vs. control; ^##^ *p* < 0.01 vs. TGF-β1 group. **(E) **The protein expression of NF-κBp65in PSCs was analyzed using Western blot, and GAPDH was used as an internal control. Relative protein abundance was quantified by densitometry analysis. Values are shown as mean ± SD (*n* = 3). *p < 0.05, **p < 0.01 vs. control; ^##^ *p* < 0.01 vs. TGF-β1 group. **(F)** The protein expression of TGF-βR1 and p-TAK in PSC was analyzed using Western blot, and GAPDH was used as an internal control. Relative protein abundance was quantified by densitometry analysis. Values are shown as mean ± SD (*n* = 3). **p < 0.01 vs. control; ^##^ *p* < 0.01 vs. TGF-β1 group.

To explore the concrete mechanism of baicalin inhibiting PSC activation, we further detected the alteration of NF-κB in the pancreas during the development of CP. Compared with the control group, the protein level of NF-κB p65 expression was significantly increased at 4 and 6 weeks after modeling, while the phosphorylated NF-κB p65 showed a high level much earlier and reached its peak at 4 weeks. However, after administration of baicalin, the expression of NF-κB p65 and phosphorylated NF-κB p65 decreased significantly. The result suggested that baicalin can inhibit the activation of NF-κB p65 in the pancreas (Figure 4A).

We performed ICH and found many NF-κB p65 positive cells in the pancreas at 4 and 6 weeks after modeling. The positive staining of NF-κB p65 was mainly observed in PSCs with the progress of fibrosis, and some infiltrated inflammatory cells or acinar cells were also stained (Figure 4B). To further affirm the relationship between NF-κB and PSCs, we performed the co-location of α-SMA and NF-κB in the pancreas using immunofluorescence double staining. As shown in Figure 4C, α-SMA (red) and NF-κB (green) were co-located in the pancreas. The results suggested that NF-κB in PSC was related to CP. After treatment with baicalin, the staining of both α-SMA and NF-κB significantly decreased compared with that at the same timepoint in the CP group (Figure 4C). This result implied that baicalin not only suppressed the activation of PSC in the development of CP but also inhibited NF-κB overexpression in the activated PSCs.

To further verify the effects of baicalin on PSC activity, we isolated the mouse PSCs, stimulated them with TGF-β1, and observed the alteration of PSC activity with or without baicalin. The protein level of α-SMA in PSCs was significantly increased after 6, 12, and 24 h of TGF-β1 stimulation, but the α-SMA level significantly reduced after baicalin treatment at 12 and 24 h. The results demonstrated that baicalin can inhibit the PSC activation induced by TGF-β1 (Figure 4D). As shown in Figure 4E, after stimulation with TGF-β1, PSCs showed a high level of NF-κB p65 expression at 2 h and maintain a higher status until 12 h. We also detected the effect of baicalin on NF-κB p65 expression and found that baicalin can inhibit NF-κB p65 expression in PSCs after TGF-β1 treatment. However, it is not clear by what mechanism TGF-β1 induced NF-κB p65 activation in PSCs. We hope to investigate whether TGF-β1 could accelerate its receptor and downstream signaling pathway to activate NF-κB p65. We found that the levels of TGF-βR1 and p-TAK in PSCs were remarkably higher after TGF-β1 stimulation, but both were relatively lower after baicalin treatment (Figure 4F).

### Baicalin can Reduce Macrophage Infiltration and Monocyte Chemotactic Protein-1 Secretion

**FIGURE 5:**
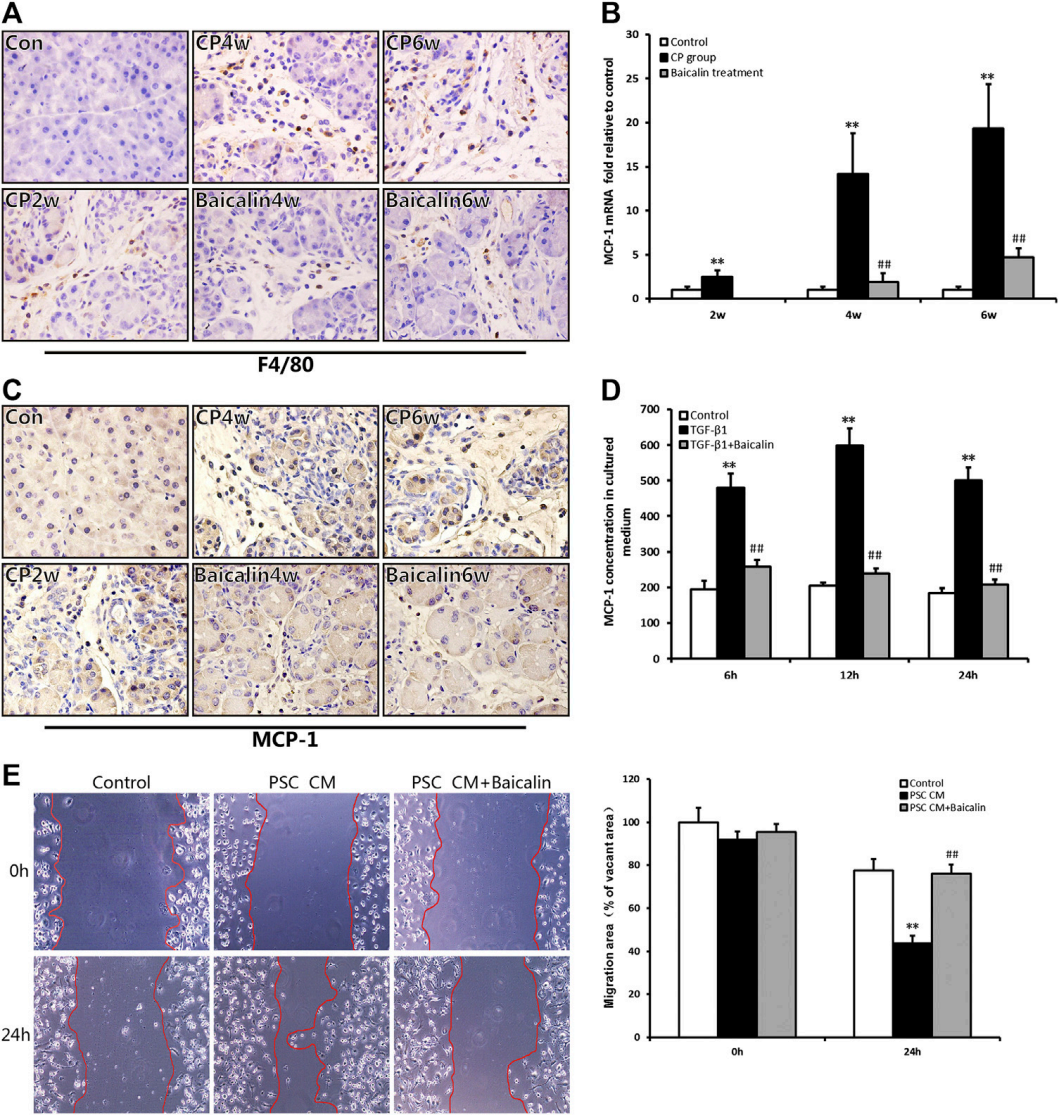
Effect of baicalin on macrophage infiltration and MCP-1 secretion. **(A)** Immunohistochemistry staining for F4/80 in different groups; the brown color shows positive cells (original magnification, × 400). **(B)** MCP-1 mRNA levels in the pancreas determined by RT-PCR. Values are shown as mean ± SD (*n* = 4). **p < 0.01 vs. control; ^##^ *p* < 0.01 vs. CP group. **(C)** Immunohistochemistry staining for MCP-1 in different groups; the brown color shows positive cells (original magnification, ×400). **(D)** MCP-1 concentrations in the PSC culture supernatant were measured by ELISA, and values are shown as mean ± SD (*n* = 3). **p < 0.01 vs. control; ^##^ *p* < 0.01 vs. TGF-β1 group. **(E)** Migration ability of macrophages in different groups was detected by the wound healing assay.

As mentioned above, pancreatic injury was accompanied by the infiltration of inflammatory cells with CP progression. We further performed IHC examination to detect the macrophage marker of F4/80 and found that F4/80-positive cells in the pancreas were significantly increased in the CP model (Figure 5A); however, the number of F4/80-positive cells, which represented macrophage infiltration, was reduced by baicalin. To further investigate the influence of chemokines on macrophage infiltration, we detected the mRNA expression in the pancreas of one of the chemokines, MCP-1, using real-time PCR. Compared with the control group, MCP-1 mRNA expression was significantly increased in the CP group at 2, 4, and 6 weeks (*p* < 0.01), but after administration of baicalin, it was remarkably decreased compared with that in CP group at the same timepoints (*p* < 0.01; Figure 5B). We then performed IHC to locate the cells that produced MCP-1 in the pancreas, and we found that the number of MCP-1-positive stained cells was remarkably increased, and baicalin treatment significantly reduced the ratio of positive-stained cells (Figure 5C). At the same time, we observed that MCP-1-positive staining cells mainly accumulated among PSC and inflammatory cells.

To confirm PSCs as the source of MCP-1, the level of MCP-1 in the supernatant of PSC was detected by ELISA *in vitro*. We found that the level of MCP-1 significantly had increased at 6, 12, and 24 h after stimulation of TGF-β1 (*p* < 0.01), however, the MCP-1 level was significantly lower after baicalin treatment (Figure 5D). This result suggested that baicalin could inhibit the release of MCP-1 from activated PSCs induced by TGF-β1. However, it is not clear whether the excessive MCP-1 contained in the supernatant of PSCs recruited macrophages from the pancreatic environment. Therefore, we observed movement of macrophages by performing wound healing assay to detect the influence of PSC supernatant on the migration of macrophages. The supernatant from TGF-β1-stimulated PSCs could significantly enhance the migration ability of macrophages compared with the control group. The movement of macrophages was remarkably inhibited by baicalin treatment (Figure 5E). All the above results indicated that baicalin can inhibit the migration of macrophages by inhibiting MCP-1 secretion from PSCs.

## Discussion

PSC activation has been regarded as the core event in the development of pancreatic fibrosis, and it is considered to be the key target for CP treatment. Recently, some Chinese herbal medicines have been used in such treatment. However, at present, there are no methods that have been proven effective for limiting the progress of pancreatic fibrosis. Yet, it has been gradually realized that some traditional Chinese herbal medicines, such as DCHD and XCHD, show significant clinical benefits in the treatment of CP, which could remarkably improve the clinical symptoms of CP patients (Liu C. et al., 2019; Cui et al., 2017; Shi et al., 2020). Moreover, similar effects have been confirmed in some animal experiments (Xu et al., 2016; Duan et al., 2017; Zhang et al., 2017). Our previous study found that DCHD can alleviate the degree of pancreatic inflammation and fibrosis in several mouse models of CP (Xu et al., 2016; Duan et al., 2017). Some experiments showed that baicalin, the main component in Baikal skullcap, itself the important herb in DCHD and XCHD, plays a significant role in anti-inflammation and anti-fibrosis (Dinda et al., 2017; Chen et al., 2019). However, it remains uncertain whether baicalin can inhibit pancreatic fibrosis and its underlying mechanism.

In this study, we induced CP mice with cerulein and treated them with baicalin to observe the effect of baicalin on pancreatic inflammation and fibrosis. Repeated intraperitoneal injection of cerulein is based on the mechanism that recurrent pancreatitis can transform into CP. This kind of CP model is easy to be induced and can mimic the pathological progression of CP in human to a great extent, and it is a recognized model in CP research (Wei et al., 2020; Klauss et al., 2020). We found that, following baicalin administration, the degree of pancreatic injury was significantly improved, with only a small amount of acinar atrophy, inflammatory cell infiltration, and collagen deposition; the COL1A1 level in the pancreas was also remarkably reduced. The results demonstrated that baicalin can prevent pancreatic fibrosis, which may be due to the inhibition of inflammatory cell infiltration and collagen production.

The essence of pancreatic fibrosis is excessive deposition of ECM; the formation of ECM is closely related to activated PSCs, and there is a positive correlation between pancreatic fibrosis and PSC activation (Bynigeri et al., 2017; Komar et al., 2017; Xue et al., 2018). In light of the unique role of activated PSCs in the pancreatic fibrosis of CP, we focused on the alteration of PSC activation in the pancreas with or without baicalin treatment by detecting the level of α-SMA, which is a surface marker of activated PSCs. We found that the expression of α-SMA in the pancreas increased after modeling of CP, but it was significantly decreased in the baicalin group. The results demonstrated that baicalin can inhibit PSC activation in the progression of CP, but its detailed mechanism is still unknown.

It is well known that PSCs are mainly activated by some cytokines released from inflammatory cells infiltrating the pancreatic microenvironment (Nielsen et al., 2017; Kusiak et al., 2020). Some reports have highlighted that, as an important transcription factor, NF-κB is closely related to the progress of pancreatic fibrosis (Chan et al., 2017; Liu et al., 2017; Chen et al., 2020). It primarily comprises a family of five Rel-domain-containing proteins, namely, NF-κB1 (p50/p105), NF-κB2 (p52/p100), RelA (p65), Re1B, and c-Rel, which exist as homo- or heterodimers (Mitchell and Carmody, 2018; Taniguchi and Karin, 2018). It has been accepted that RelA (p65) is the most common heterodimer of NF-κB and the key protein of the NF-κB signaling pathway (Chen et al., 2020).

In this study, we detected the level of NF-κB p65 and phosphorylated p65in the pancreas, and we found that both were significantly increased after modeling CP. Treiber et al. (Treiber et al., 2011) induced a CP model in mice with RelA knockout specifically in pancreatic acinar cells (Rela^Δpanc^), and they discovered more serious manifestations in Rela^Δpanc^ mice with extensive acinar cell necrosis and pancreatic fibrosis; meanwhile, the expressions of α-SMA and F4/80 in pancreas were also increased. Thus, it is clear that the NF-κB signaling pathway in acinar cells is closely related to pancreatic inflammation and fibrosis. However, the specific role of the NF-κB signaling pathway in PSCs has not been further studied.

To identify the source of the high expression of NF-κB in the pancreas after modeling, we performed IHC staining and found large numbers of NF-κB-positive staining cells that focused on PSCs, some infiltrated inflammatory cells, and acinar cells. Further immunofluorescence double staining showed many co-localizations of NF-κB and α-SMA in the pancreas of CP mice. These results revealed that NF-κB in PSCs was not only activated and participated in the progress of CP but may also be a potential target for CP treatment. As expected, the levels of NF-κB and co-expression of NF-κB and α-SMA in the pancreas of the Baicalin group were significantly decreased. This suggested that baicalin may inhibit PSC activation by downregulating the NF-κB of PSCs.

TGF-β is one of the strongest fibrogenic cytokines, and it is involved in the fibrogenesis of many diseases (Meng et al., 2016; Kim et al., 2018; Ma and Meng, 2019). During CP progression, upregulation of TGF-β in the pancreas further activates PSCs, promotes the synthesis of the ECM, and accelerates pancreatic fibrosis (Liu P. et al., 2019; Sun et al., 2018; Xu et al., 2018). To explore the detailed mechanism of baicalin inhibiting PSC activation, we further isolated the primary PSCs of mice, cultured them to the second generation, and stimulated them with TGF-β1. We found that PSCs were activated via overexpression of α-SMA; the levels of TGF-βR1, p-TAK1, and NF-κB were also increased, suggesting that the detailed mechanism of TGF-β1 activating PSCs may be as follows: TGF-β1 binds to TGF-βR1 on PSCs, which causes TAK phosphorylation, further leading to NF-κB activation (Won et al., 2016). After baicalin treatment, the expression of α-SMA in PSC decreased; all other indicators, including TGF-βR1, p-TAK1, and NF-κB, were also decreased. Our results clarified that baicalin can inhibit the activation of PSCs by downregulating the TGF-β1/TGF-βR1/TAK1/NF-κB signaling pathway in PSCs.

Based on the close relationship between inflammation and fibrosis progression, some research has demonstrated that proinflammatory cytokines can induce PSC activation, while activated PSCs can further secrete more inflammatory factors, such as MCP-1, which in turn, accelerate PSC activation (Gao et al., 2013). In our study, we found that macrophage infiltration in the pancreas increased, and MCP-1 mRNA was also overexpressed with the development of CP. Moreover, IHC staining of MCP-1 showed that PSCs are the main positive cells in the pancreas. Further *in vitro* experiments proved that MCP-1 in PSC supernatant increased significantly after TGF-β1 stimulation, and PSC may be an important source of MCP-1 production.

As the main member of the chemokine family, MCP-1 can recruit inflammatory cells from peripheral blood to the inflammatory site, which plays a key role in the recruitment of monocytes (Deshmane et al., 2009). However, it is unknown whether the high level of MCP-1 takes part in the macrophage infiltration in the pancreas of CP and what effects of MCP-1 in PSC supernatant on macrophage migration. We further isolated macrophages from bone marrow and tested their migration ability via the wound healing assay. The results showed that the supernatant of PSCs with a high concentration of MCP-1 could remarkably increase the migration ability of macrophages, but baicalin could inhibit macrophage migration by reducing MCP-1 levels in the PSC supernatant. Similarly, *in vivo*, after treatment with baicalin, the expression of MCP-1 and F4/80 in the pancreas of CP mice both decreased significantly, suggesting that baicalin can reduce macrophage infiltration by inhibiting MCP-1 secretion. The above results indicate that activated PSCs will aggravate pancreatitis by secreting large amounts of MCP-1 to recruit more macrophages to the pancreas, while baicalin can inhibit the activation of PSCs via reducing the release of MCP-1 and macrophage infiltration.

Some reports have shown that the secretion of MCP-1 is closely related to the activation of NF-κB (Masamune et al., 2002). A study by Andoh et al. (Andoh et al., 2000) confirmed that myofibroblasts around pancreatic acini could secrete MCP-1. After stimulation using inflammatory factors, such as IL-1β and TNF-α, along with the increase of MCP-1, NF-κB was also activated. However, adding pyrrolidinedithiocarbamate (PDTC; an inhibitor of NF-κB) leads to downregulated MCP-1 levels. Combined with our findings, baicalin may reduce the secretion of MCP-1 from PSCs and inhibit the recruitment effect on macrophages. Its mechanism is mediated by regulating the NF-κB signaling pathway of PSCs.

## Conclusion

**FIGURE 6:**
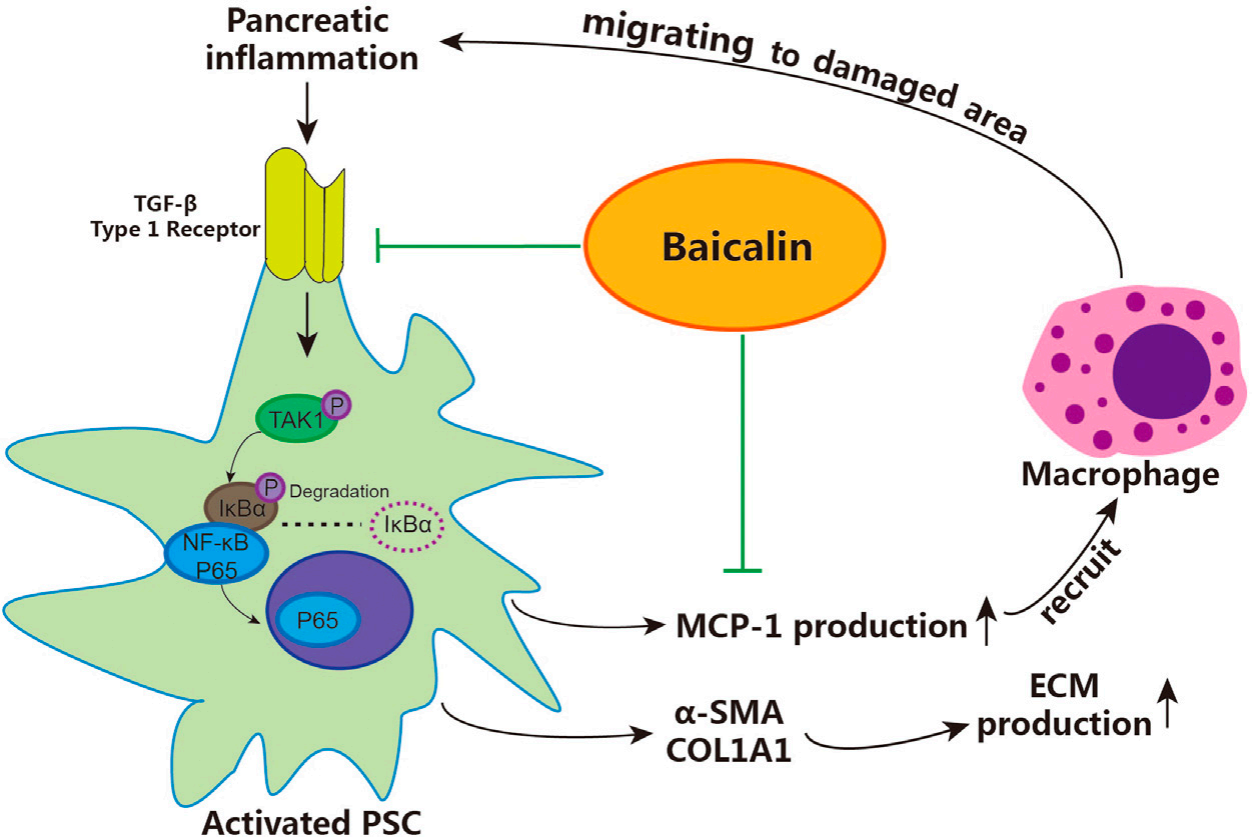
The detailed mechanism of baicalin inhibiting pancreatic fibrosis in CP. PSC: pancreatic stellate cells, ECM: extracellular matrix.

According to the above results, we demonstrated that baicalin can directly inhibit the activation of PSCs by downregulating the activity of the TGF-β1/TGF-βR1/TAK1/NF-κB signaling pathway, achieving the effect of inhibiting pancreatic fibrosis. Moreover, baicalin can also reduce the recruitment of macrophages via inhibiting the release of MCP-1 from PSCs, then slow down the progression of CP by curbing the vicious cycle of inflammation–fibrosis (shown in Figure 6). These findings contribute to our understanding of the detailed mechanisms related to the role of activated PSCs in pancreatic fibrosis and provide a reliable theoretical basis and experimental data for using baicalin in the treatment of CP.

## Data Availability Statement

The original contributions presented in the study are included in the article/Supplementary Material, further inquiries can be directed to the corresponding authors.

## Ethics Statement

The animal study was reviewed and approved by “The Guide for Care and Use of Laboratory Animals” issued by Shaanxi University of Traditional Chinese Medicine (SUCMLAEC 2018010, Xianyang, China).

## Author Contributions

HZ and CZ designed and conducted the research; JF and LD contributed equally to the work, and both performed most of the experiments, wrote the paper and analyzed the data; NW, XX, JX and SJ performed part of the experiments. All authors approved the final manuscript.

## Funding

The study was supported by the National Natural Science Foundation of China (81673816); the special support project for high level talents in Shaanxi Province(303/141020047); the subject Innovation Team of Shaanxi University of Chinese Medicine (2019-LY14); the Natural Science Foundation of Shaanxi Province (2017JM8076).

## Conflict of Interest

The authors declare that the research was conducted in the absence of any commercial or financial relationships that could be construed as a potential conflict of interest.
