# Supplementary figures and images for: Baicalin Ameliorates Pancreatic Fibrosis by Inhibiting the Activation of Pancreatic Stellate Cells in Mice with Chronic Pancreatitis
Source: Front Pharmacol. 2021 Jan 18;11:607133. doi: 10.3389/fphar.2020.607133 (PMC7848203; doi:10.3389/fphar.2020.607133)

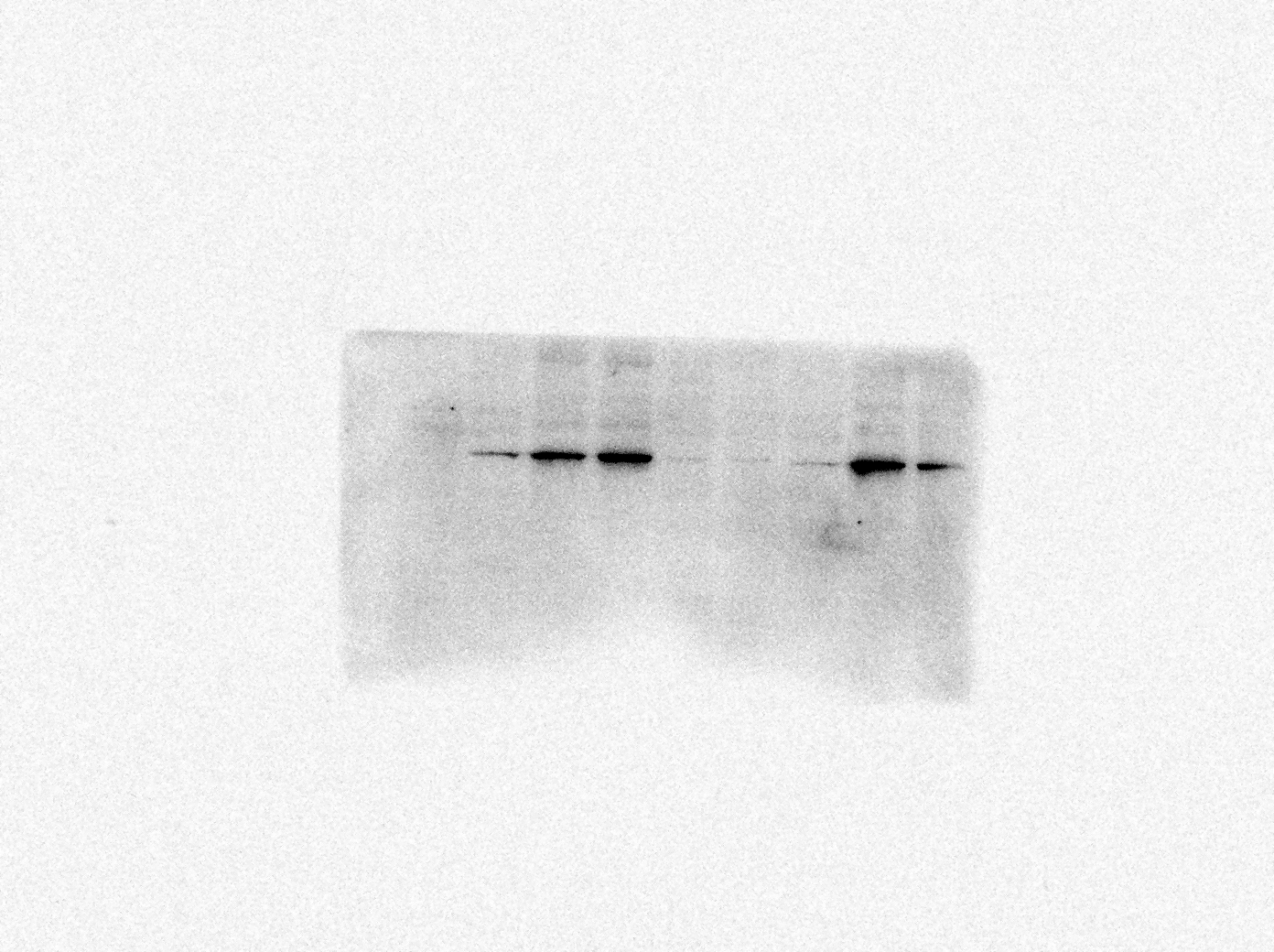

Supplement: Supplementary file 1 [file datasheet1.zip › Orginal image of Western blotting/figure 2 (D)/figure 2 (D)-COL1A1.tif]

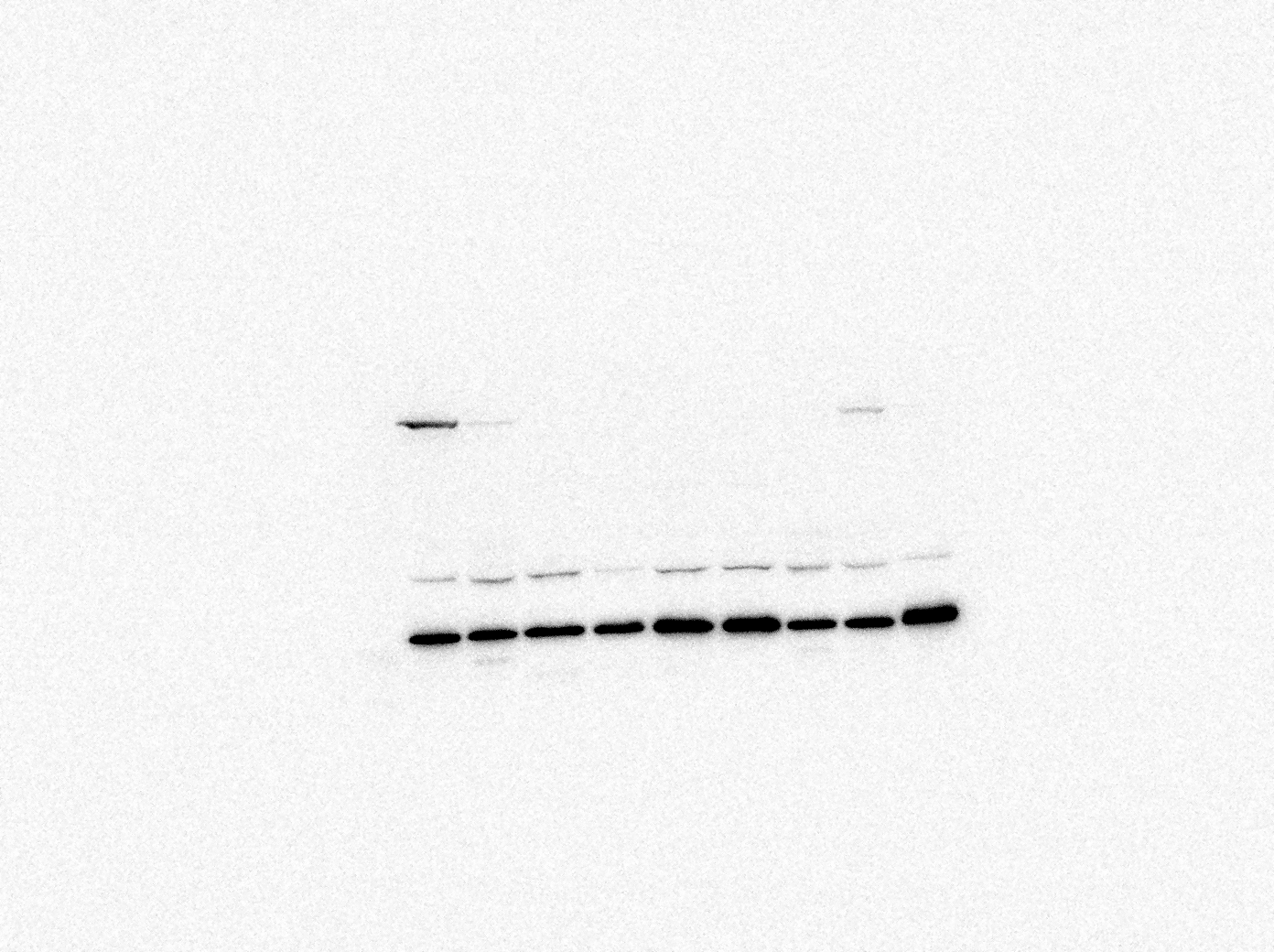

Supplement: Supplementary file 1 [file datasheet1.zip › Orginal image of Western blotting/figure 2 (D)/figure 2 (D)-GAPDH.tif]

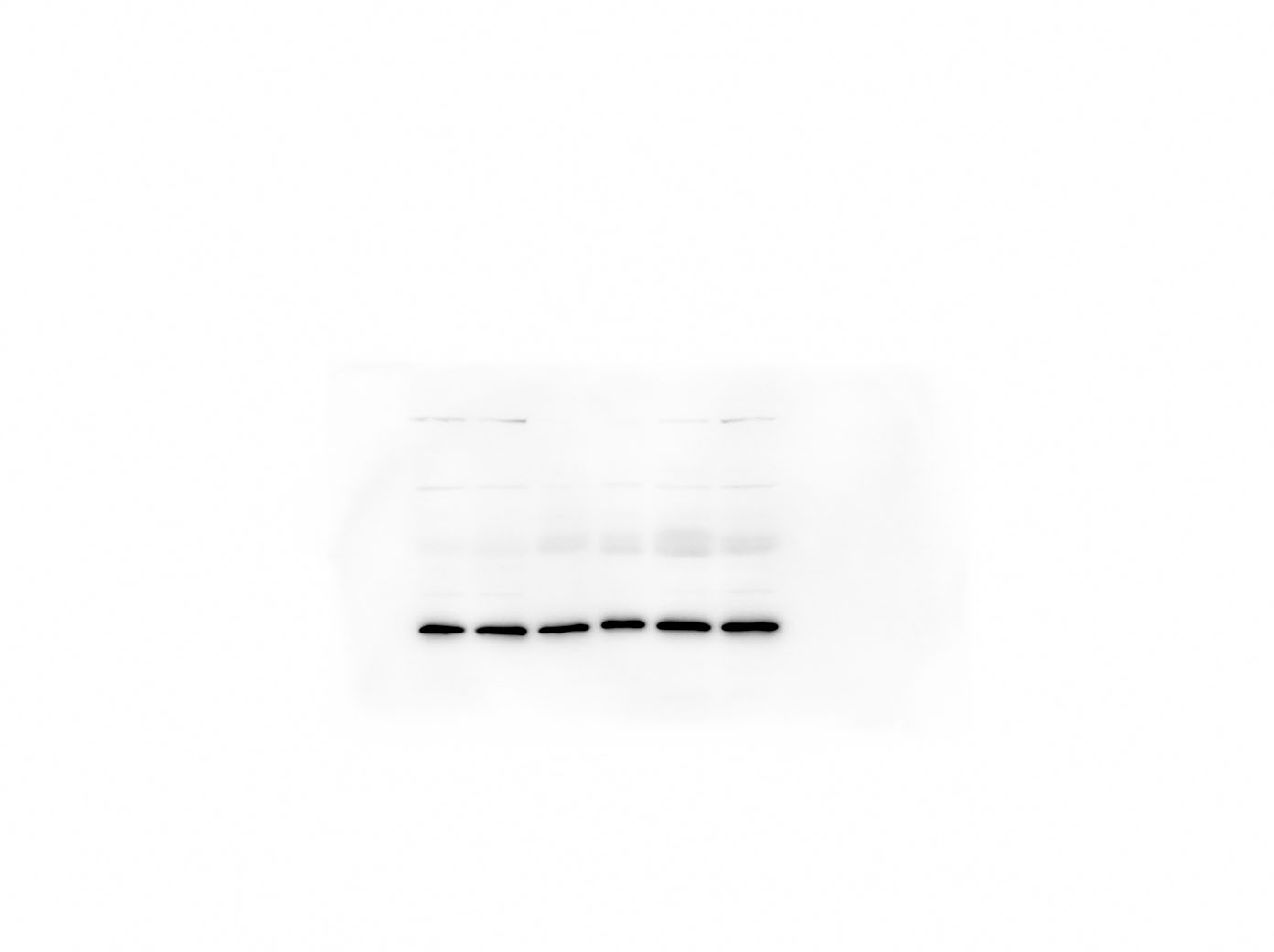

Supplement: Supplementary file 1 [file datasheet1.zip › Orginal image of Western blotting/figure 3 (B)/figure 3 (B)-GAPDH.jpg]

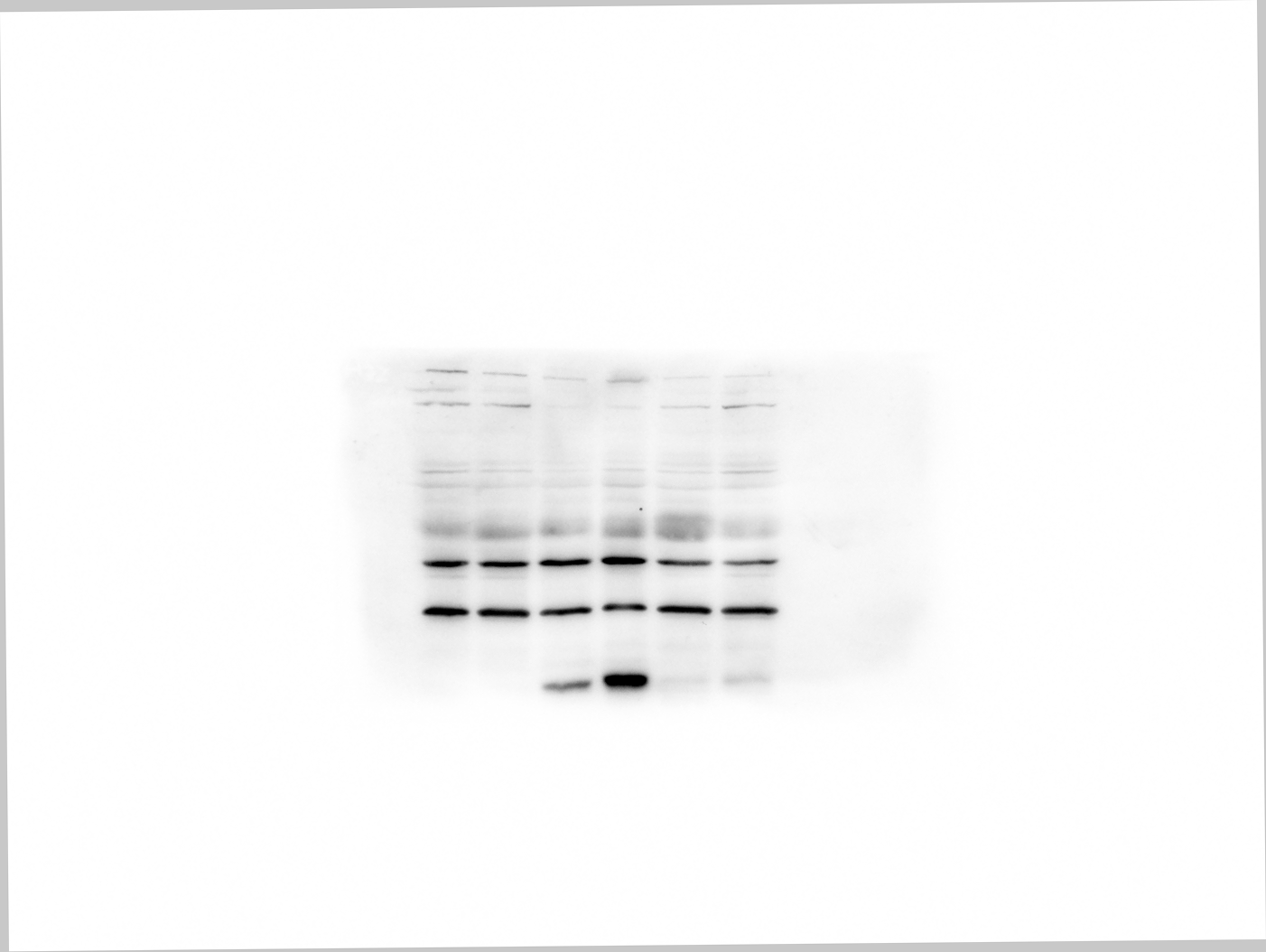

Supplement: Supplementary file 1 [file datasheet1.zip › Orginal image of Western blotting/figure 3 (B)/figure 3 (B)-a┴SMA.tif]

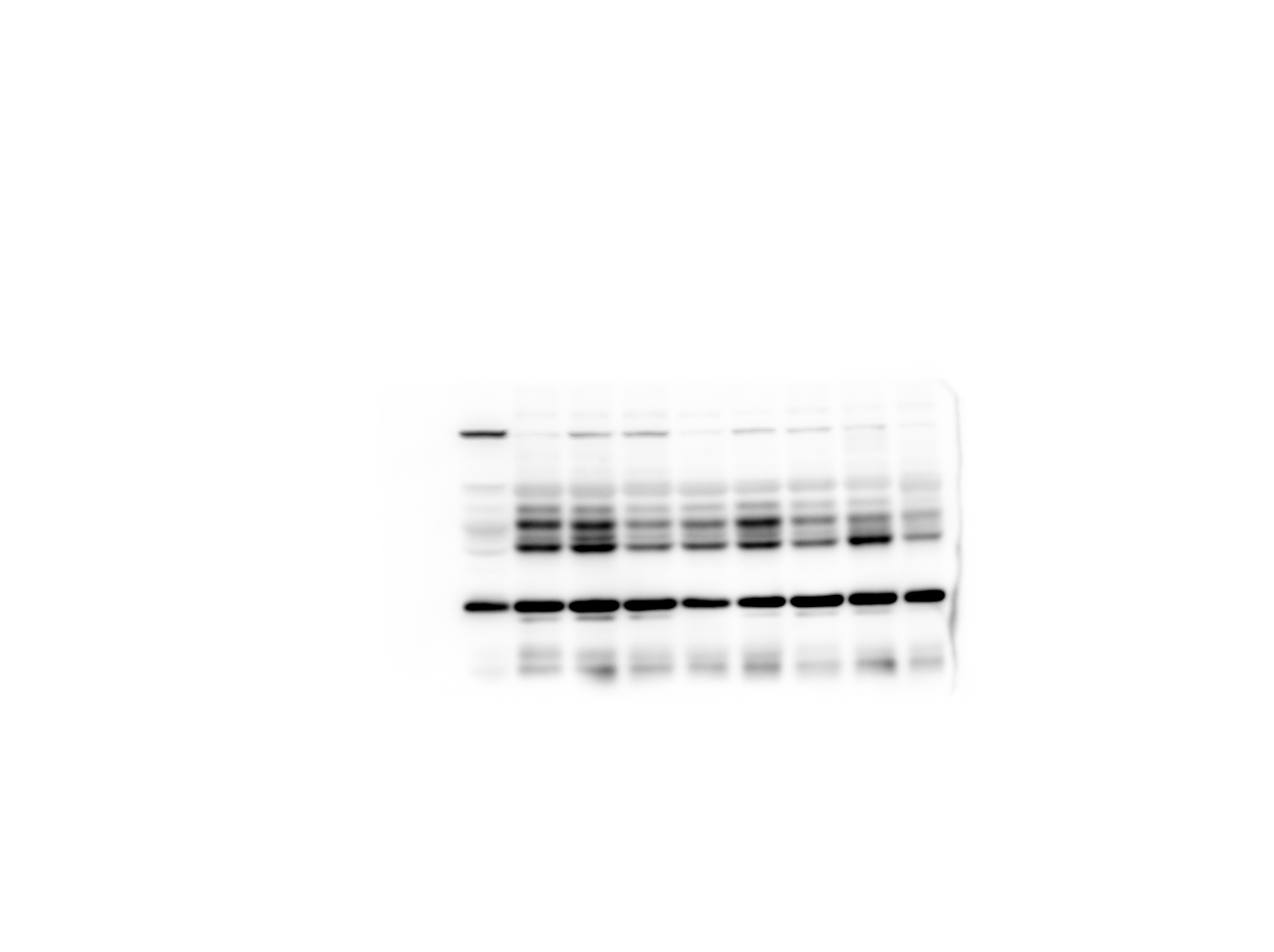

Supplement: Supplementary file 1 [file datasheet1.zip › Orginal image of Western blotting/figure 4 (A)/figure 4 (A)-GAPDH.tif]

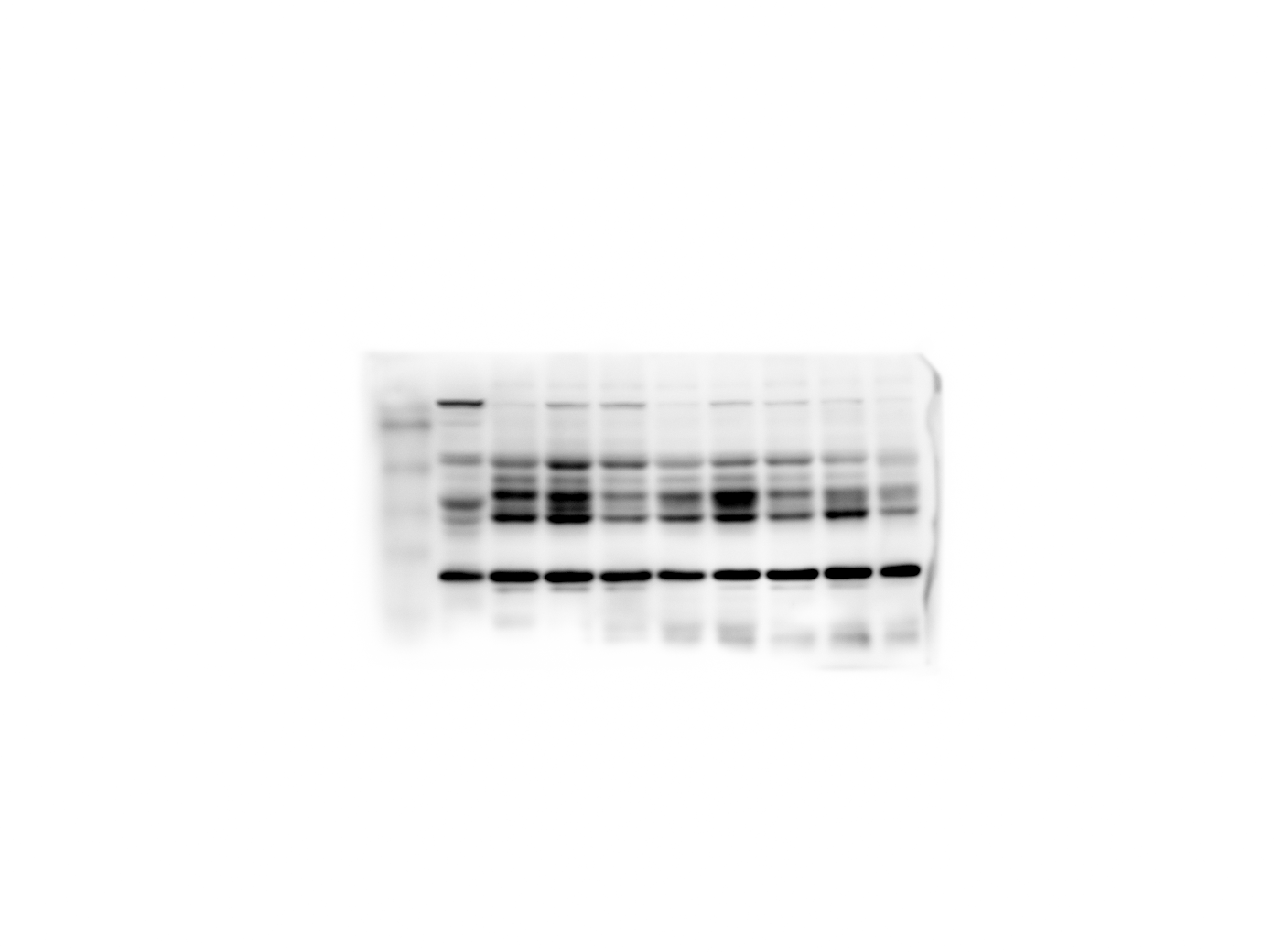

Supplement: Supplementary file 1 [file datasheet1.zip › Orginal image of Western blotting/figure 4 (A)/figure 4 (A)-p-p65.tif]

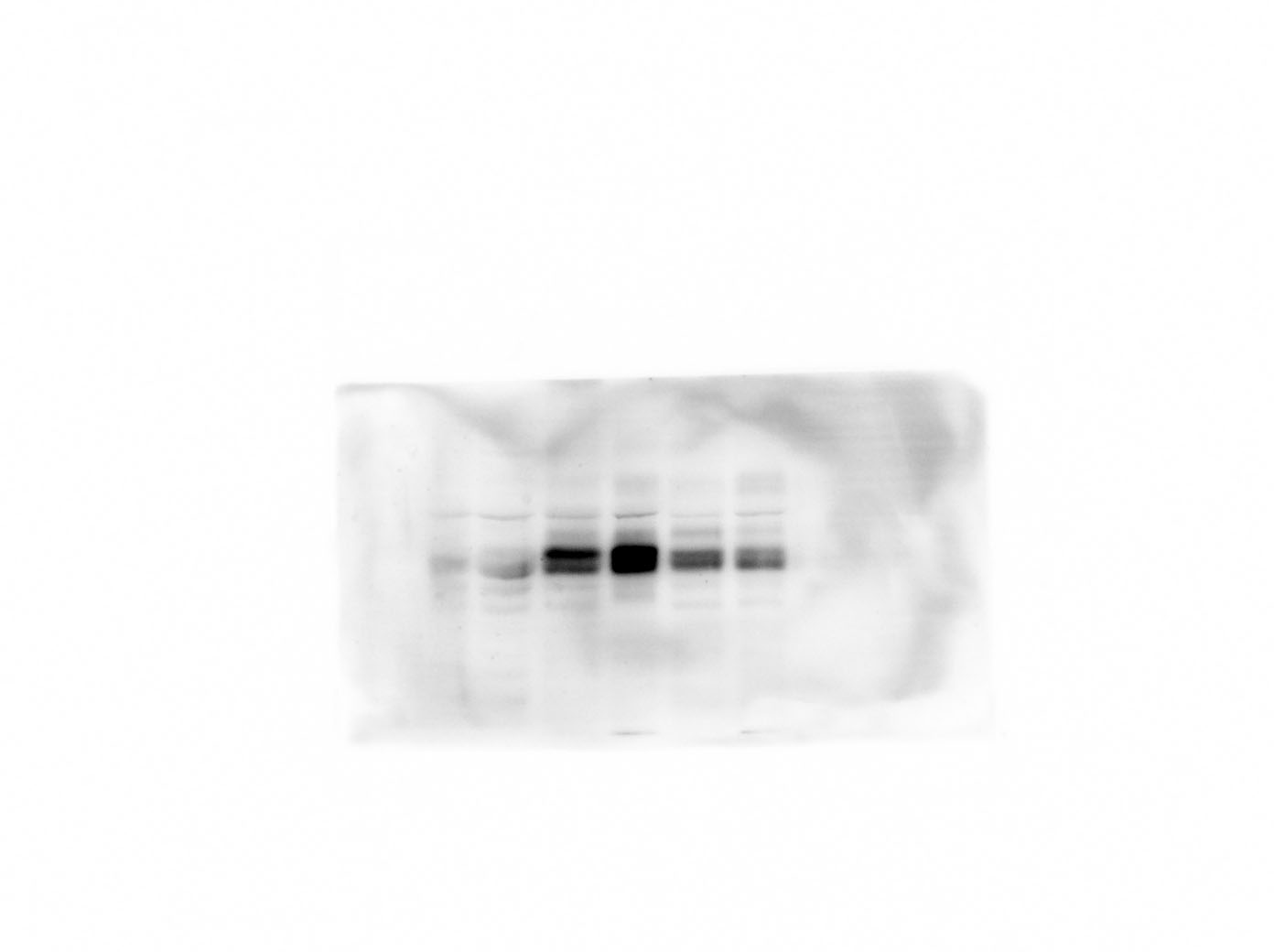

Supplement: Supplementary file 1 [file datasheet1.zip › Orginal image of Western blotting/figure 4 (A)/figure 4 (A)-p65.tif]

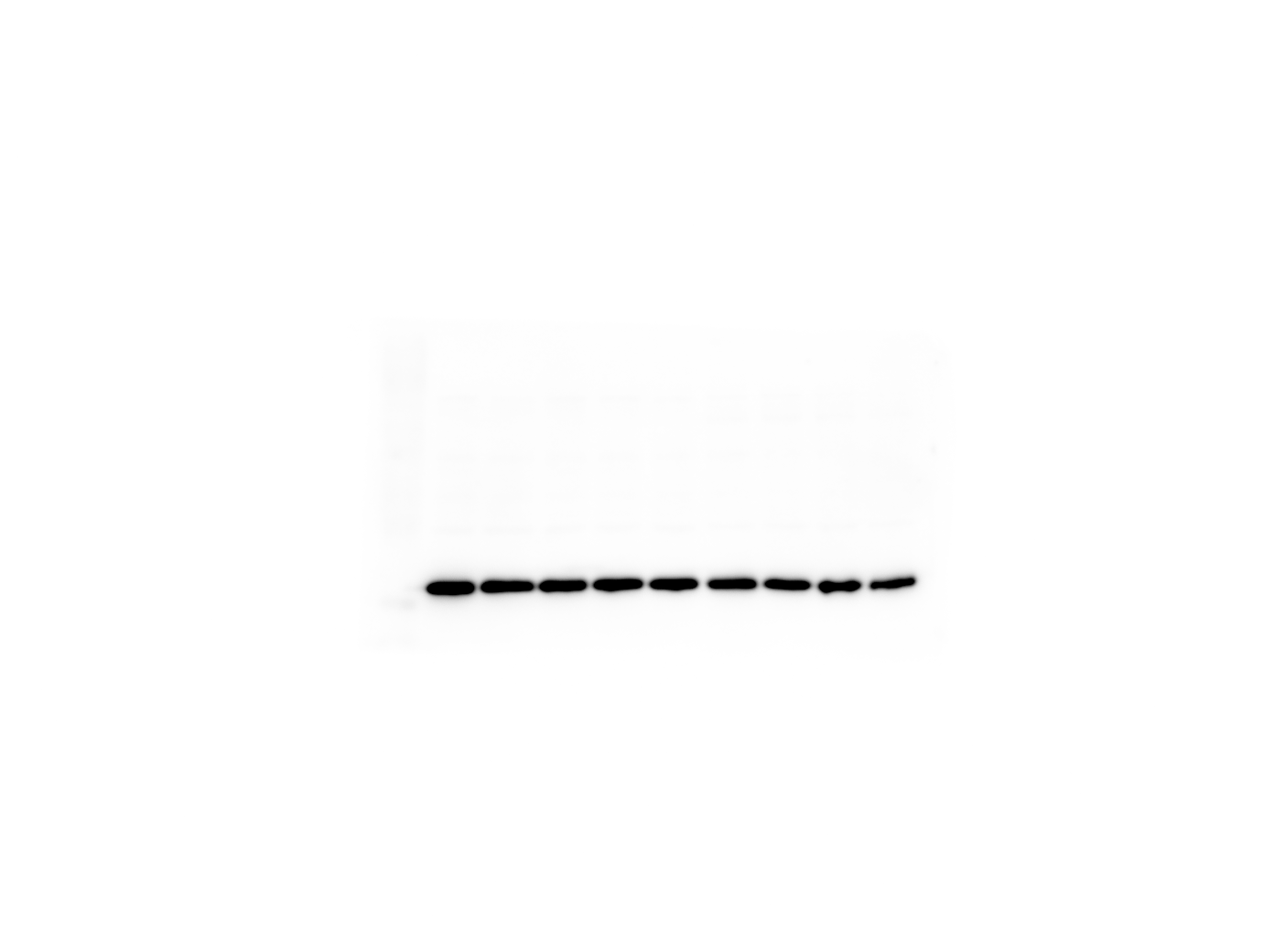

Supplement: Supplementary file 1 [file datasheet1.zip › Orginal image of Western blotting/figure 4 (D)/figure 4 (D)-GAPDH.tif]

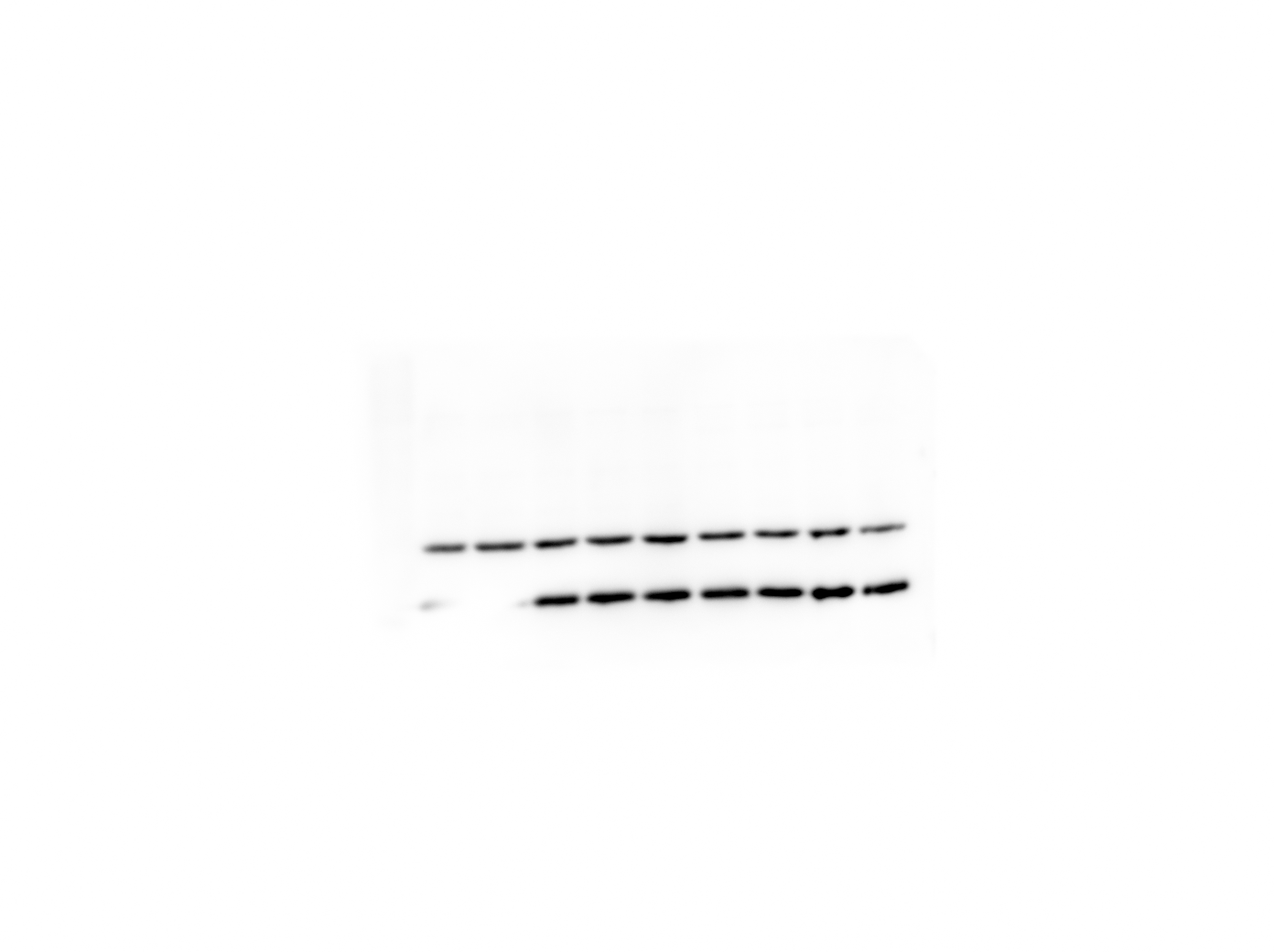

Supplement: Supplementary file 1 [file datasheet1.zip › Orginal image of Western blotting/figure 4 (D)/figure 4 (D)-a┴SMA.tif]

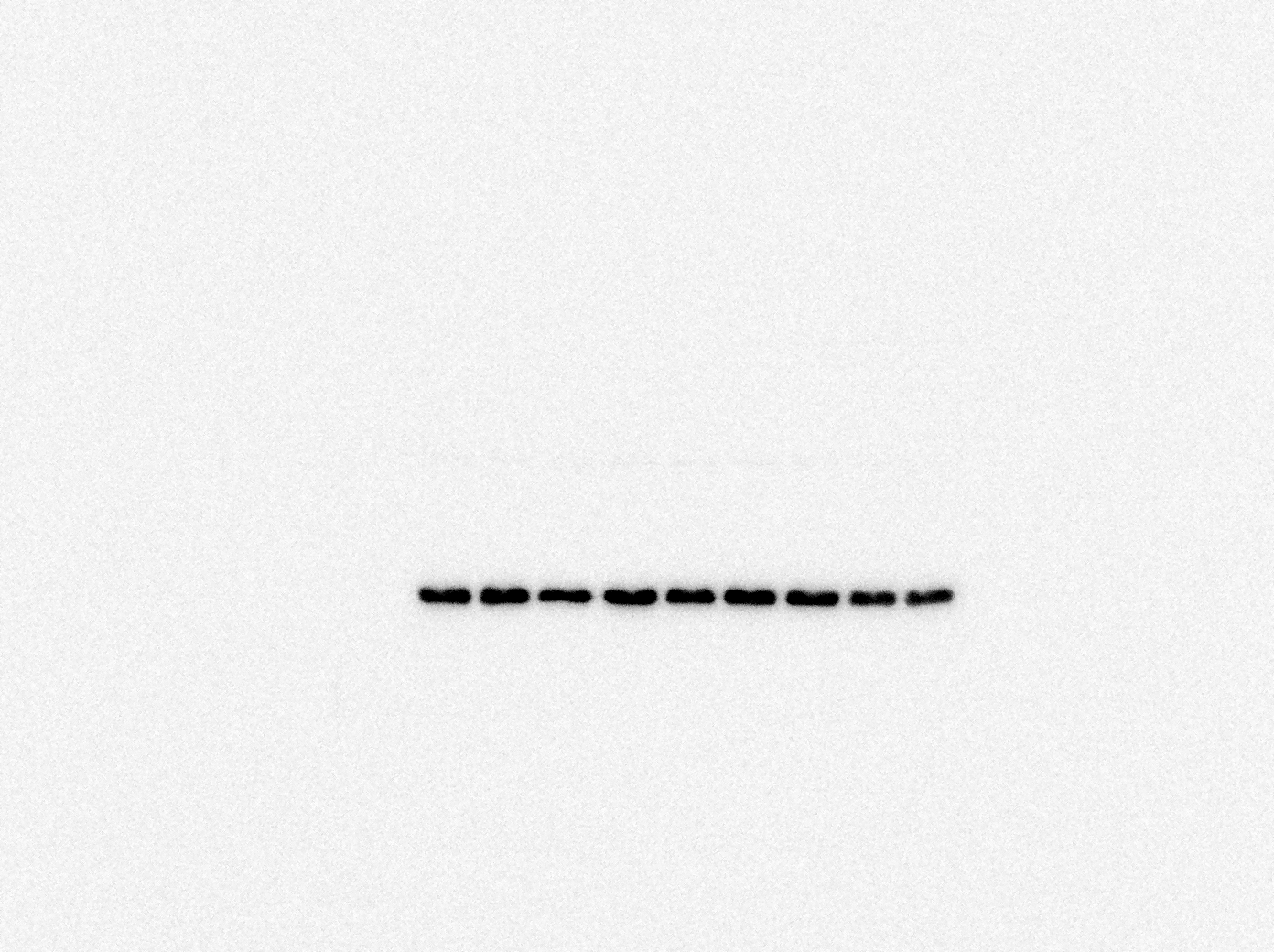

Supplement: Supplementary file 1 [file datasheet1.zip › Orginal image of Western blotting/figure 4 (E)/figure 4 (E)-GAPDH.tif]

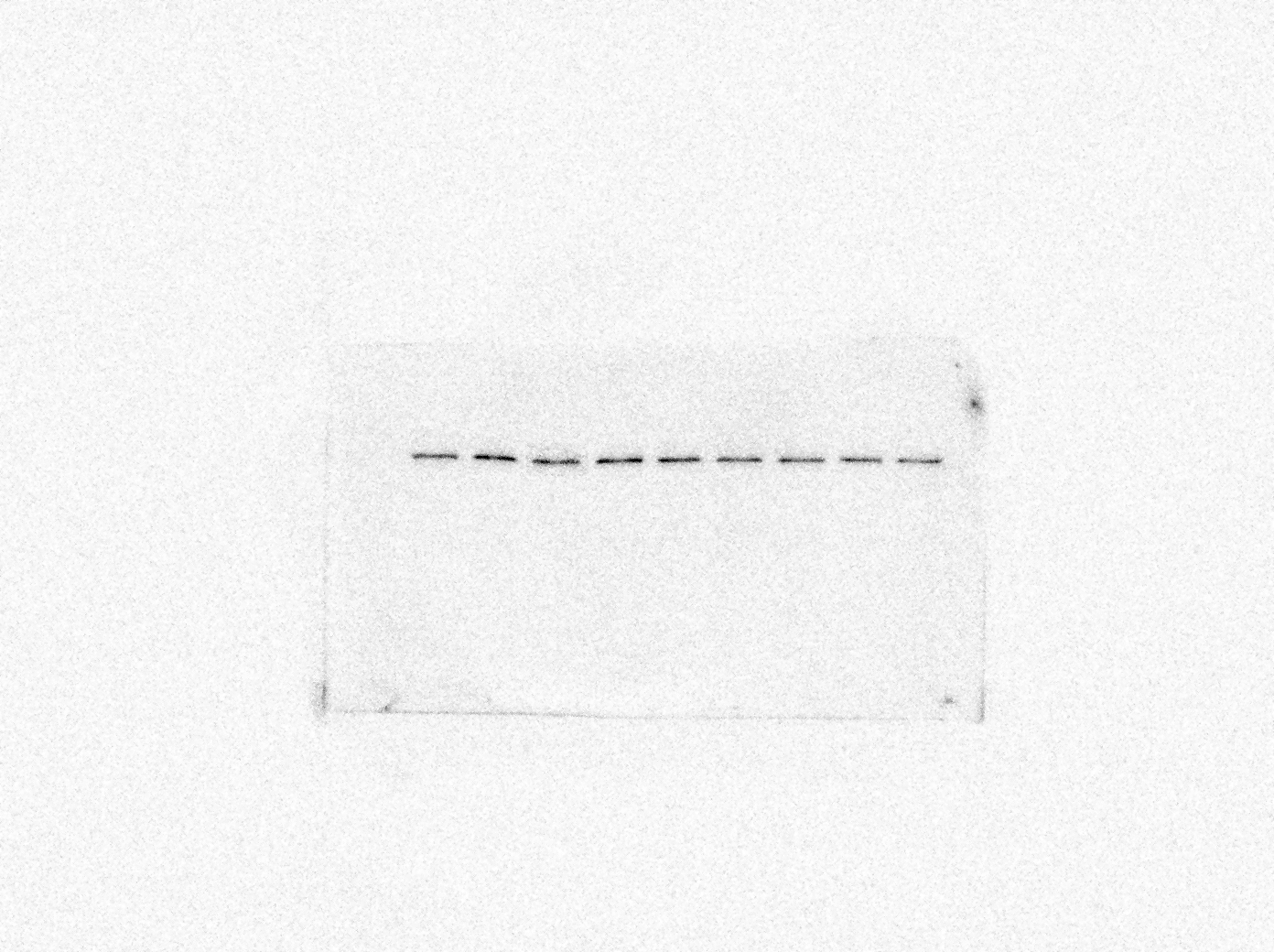

Supplement: Supplementary file 1 [file datasheet1.zip › Orginal image of Western blotting/figure 4 (E)/figure 4 (E)-p65.tif]

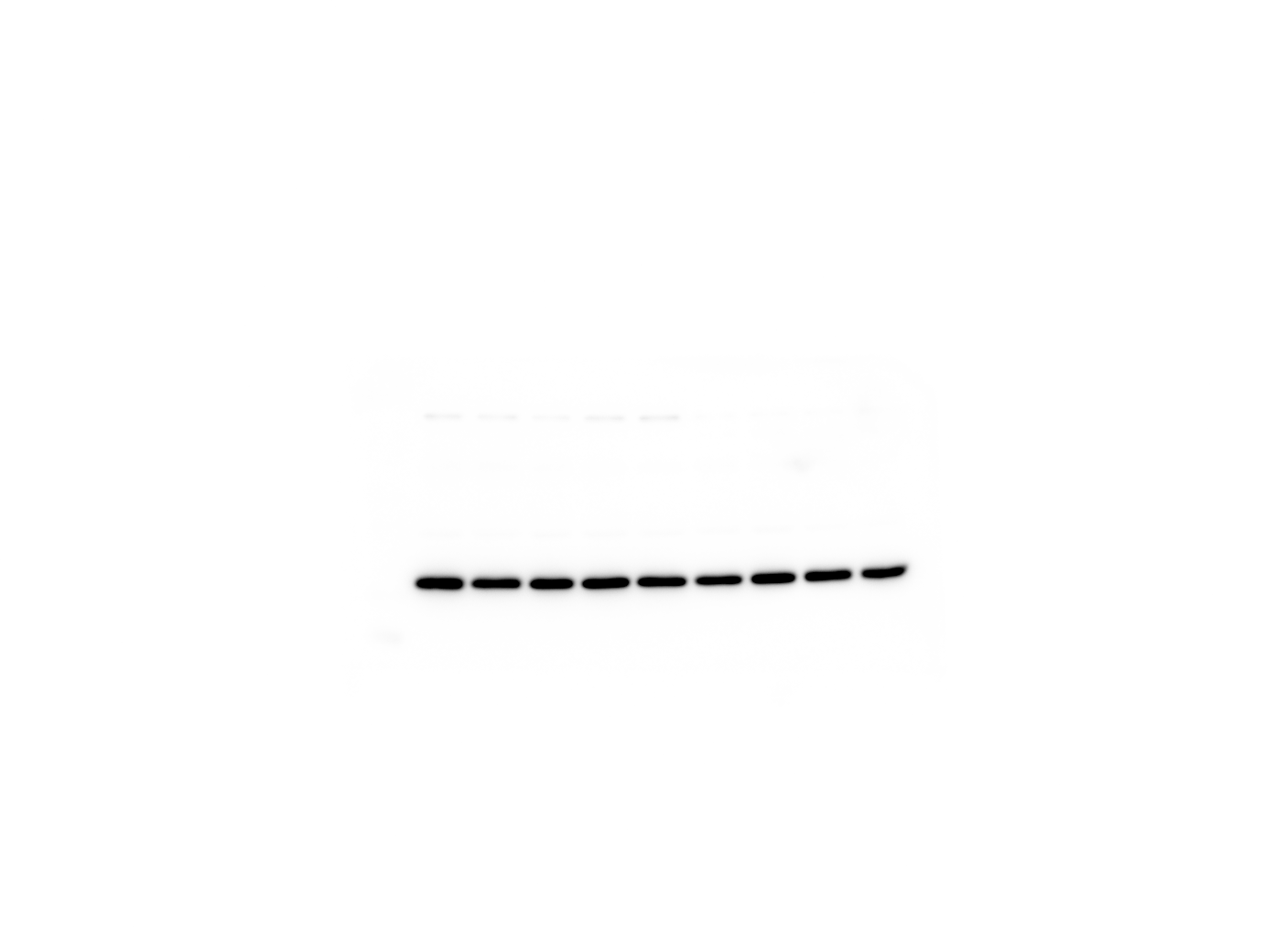

Supplement: Supplementary file 1 [file datasheet1.zip › Orginal image of Western blotting/figure 4 (F)/figure 4 (F)-GAPDH.tif]

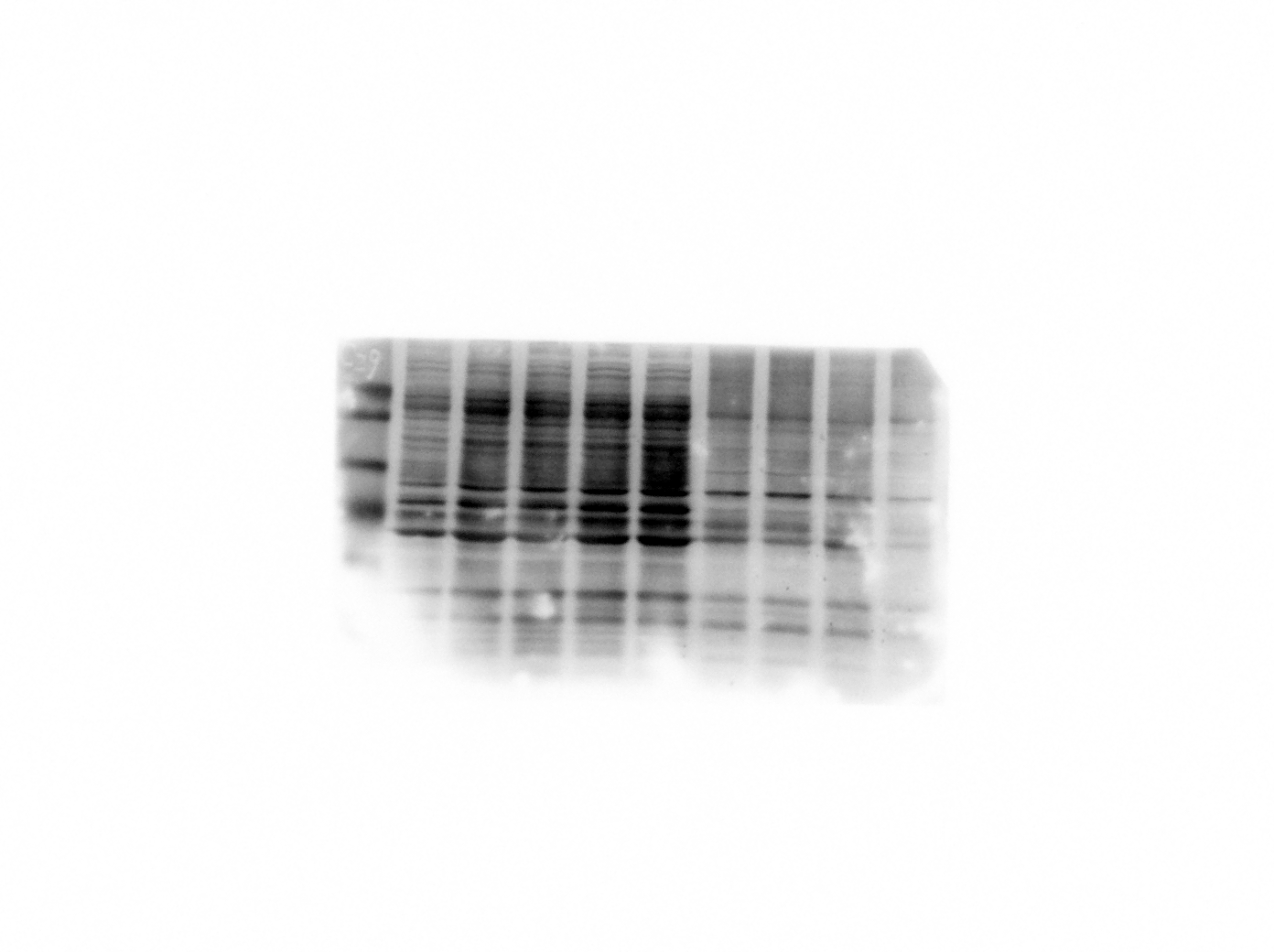

Supplement: Supplementary file 1 [file datasheet1.zip › Orginal image of Western blotting/figure 4 (F)/figure 4 (F)-p-TAK1.tif]

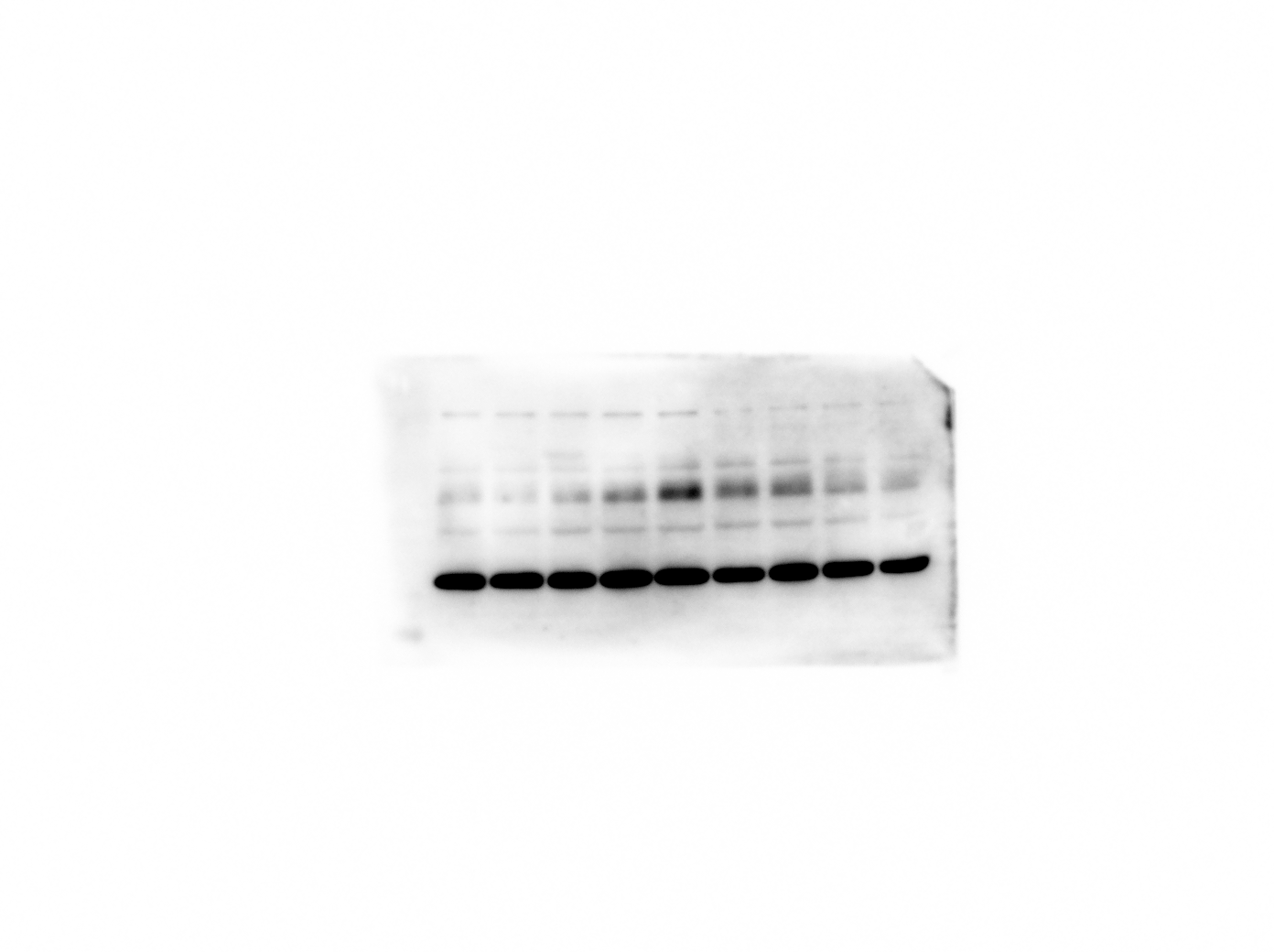

Supplement: Supplementary file 1 [file datasheet1.zip › Orginal image of Western blotting/figure 4 (F)/figure 4 (F)-TGF-a┬R1.tif]

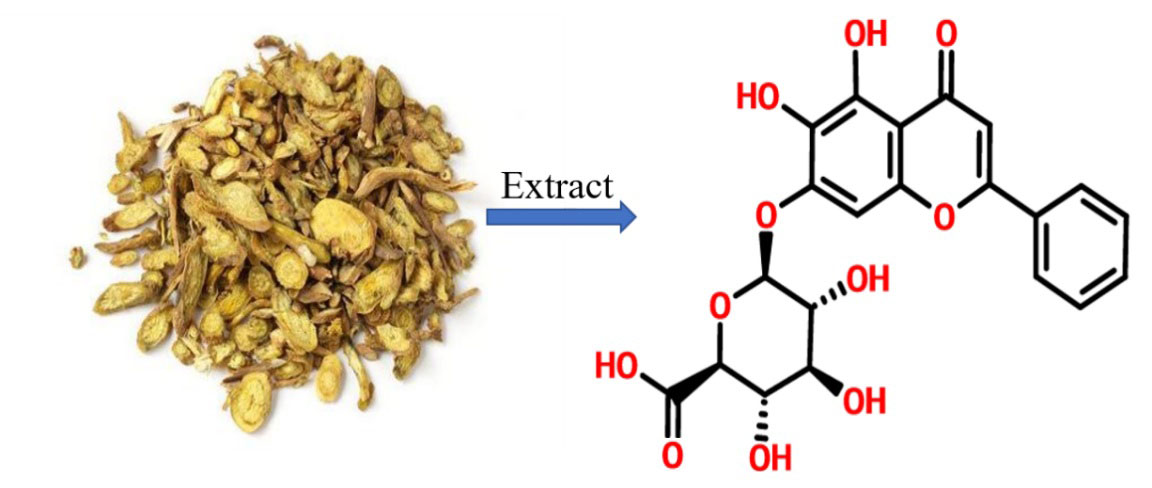

Supplement: Supplementary file 2 [file datasheet2.zip › figures-revised/figure 1/Figure1.jpg]

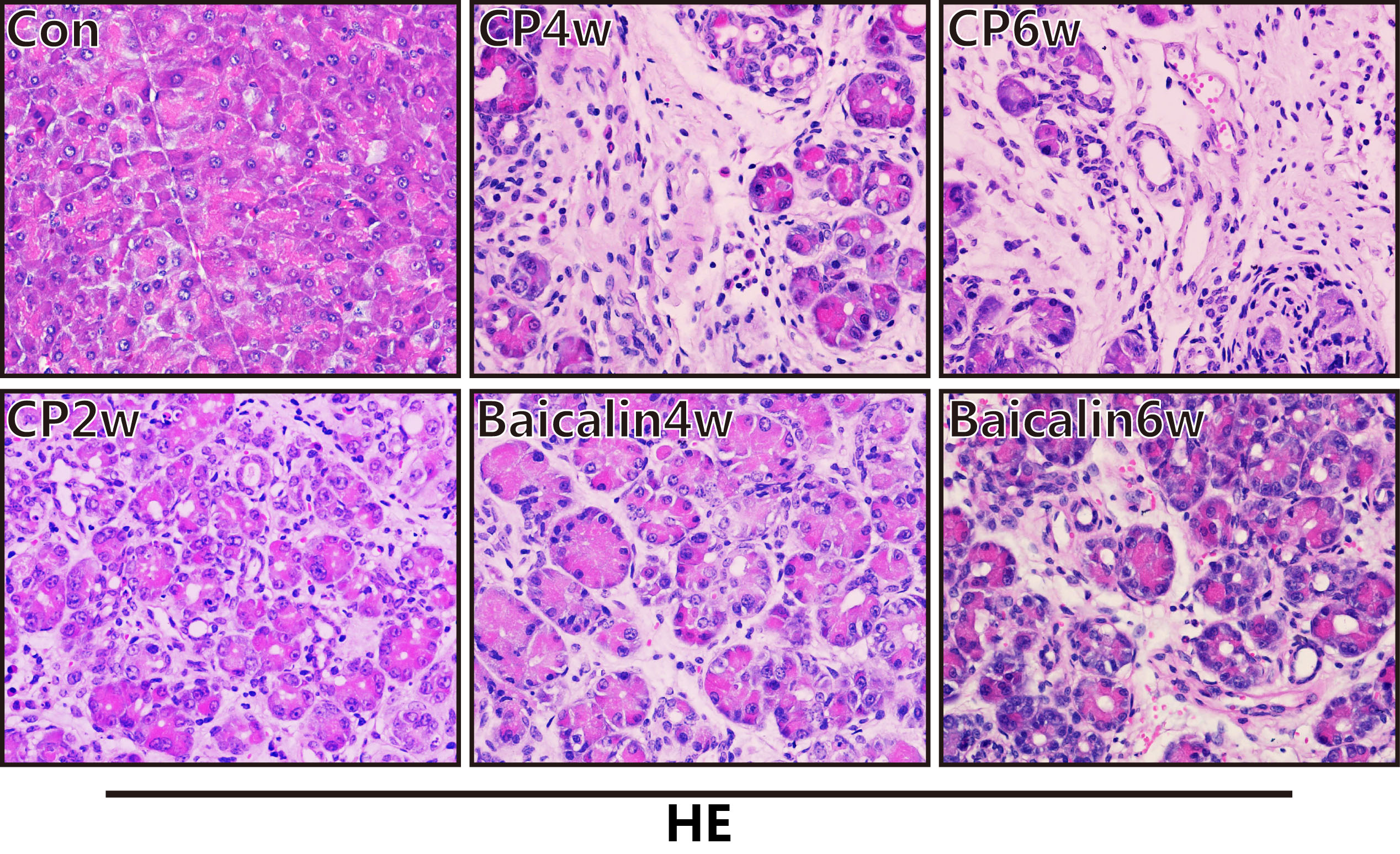

Supplement: Supplementary file 2 [file datasheet2.zip › figures-revised/figure 2/Figure 2 (A).jpg]

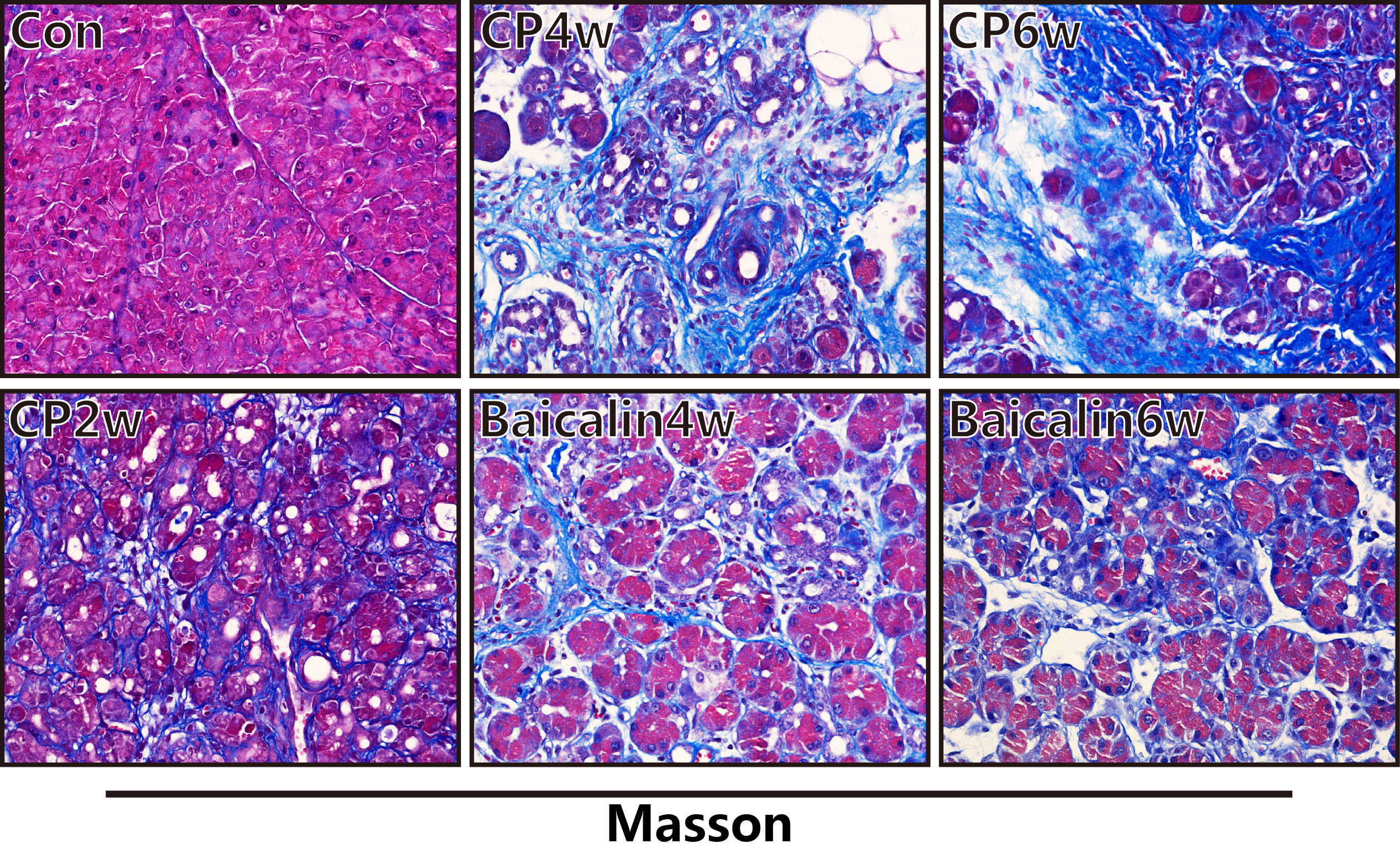

Supplement: Supplementary file 2 [file datasheet2.zip › figures-revised/figure 2/Figure 2 (B).jpg]

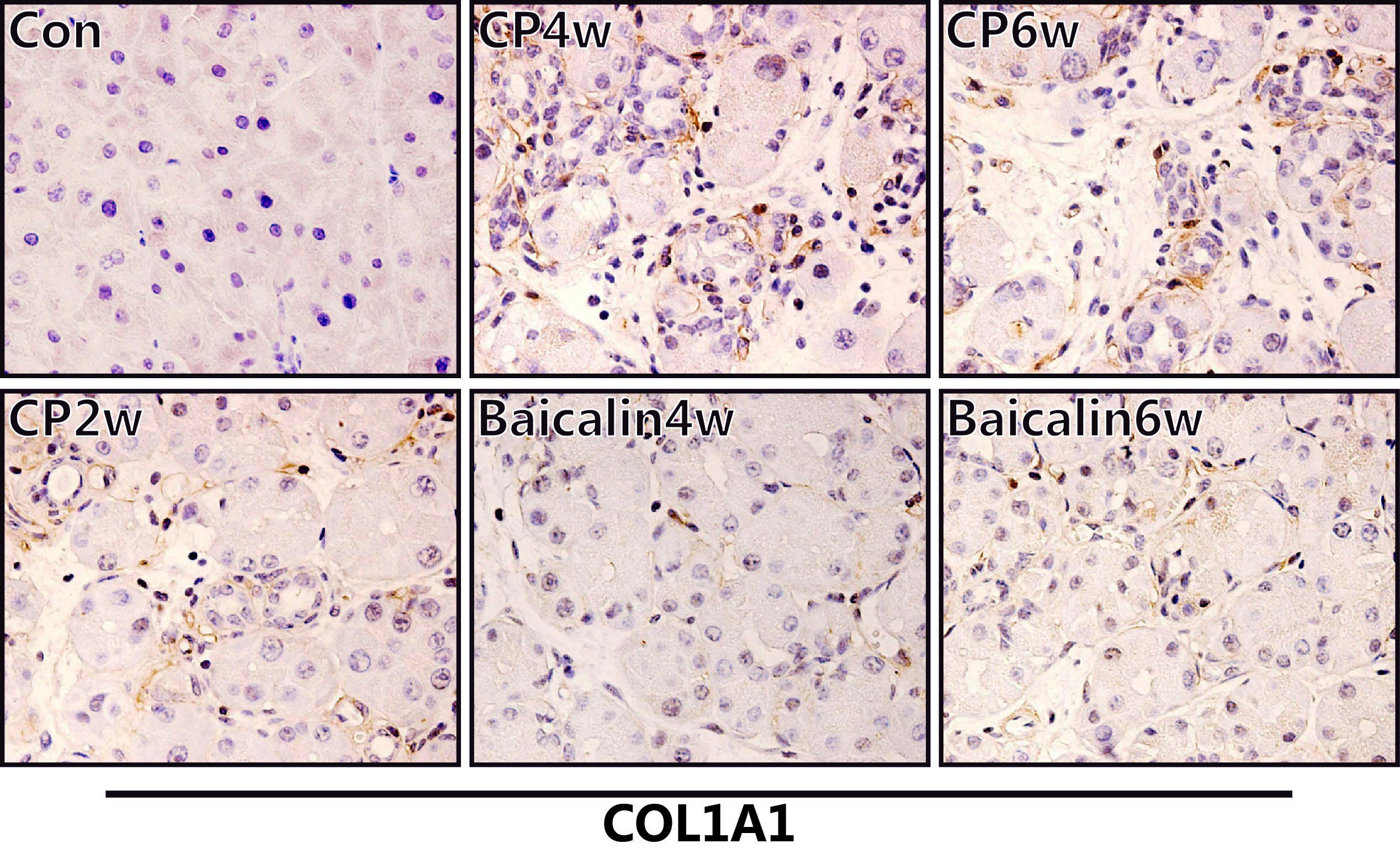

Supplement: Supplementary file 2 [file datasheet2.zip › figures-revised/figure 2/figure 2 (C).jpg]

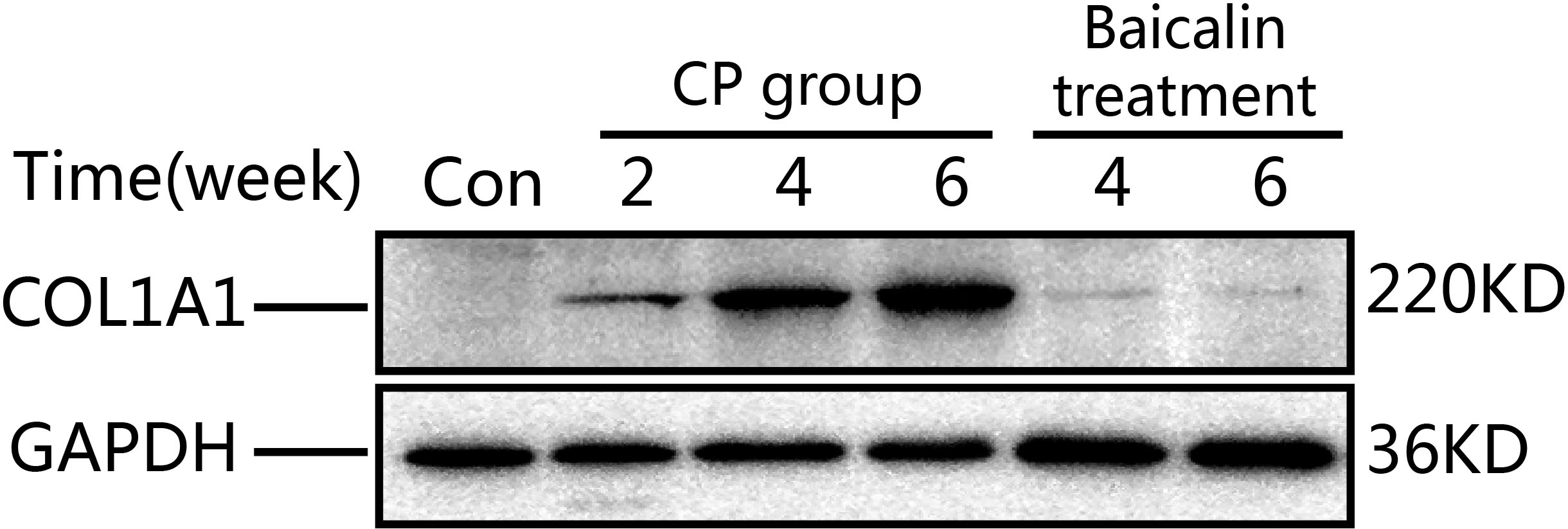

Supplement: Supplementary file 2 [file datasheet2.zip › figures-revised/figure 2/figure 2 (D)-1.jpg]

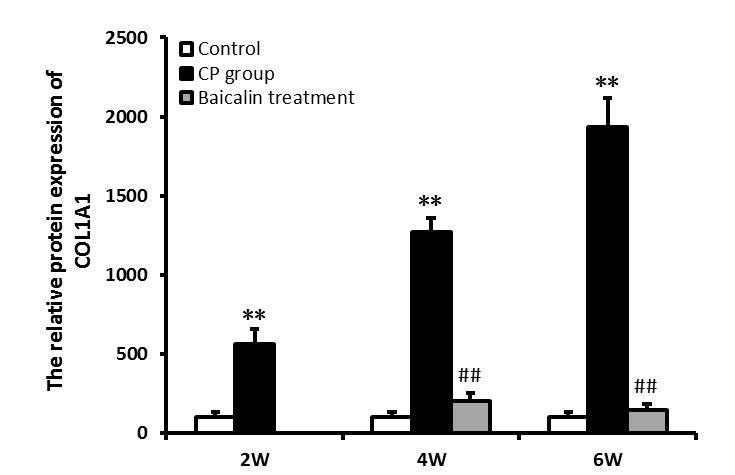

Supplement: Supplementary file 2 [file datasheet2.zip › figures-revised/figure 2/figure 2 (D)-2.jpg]

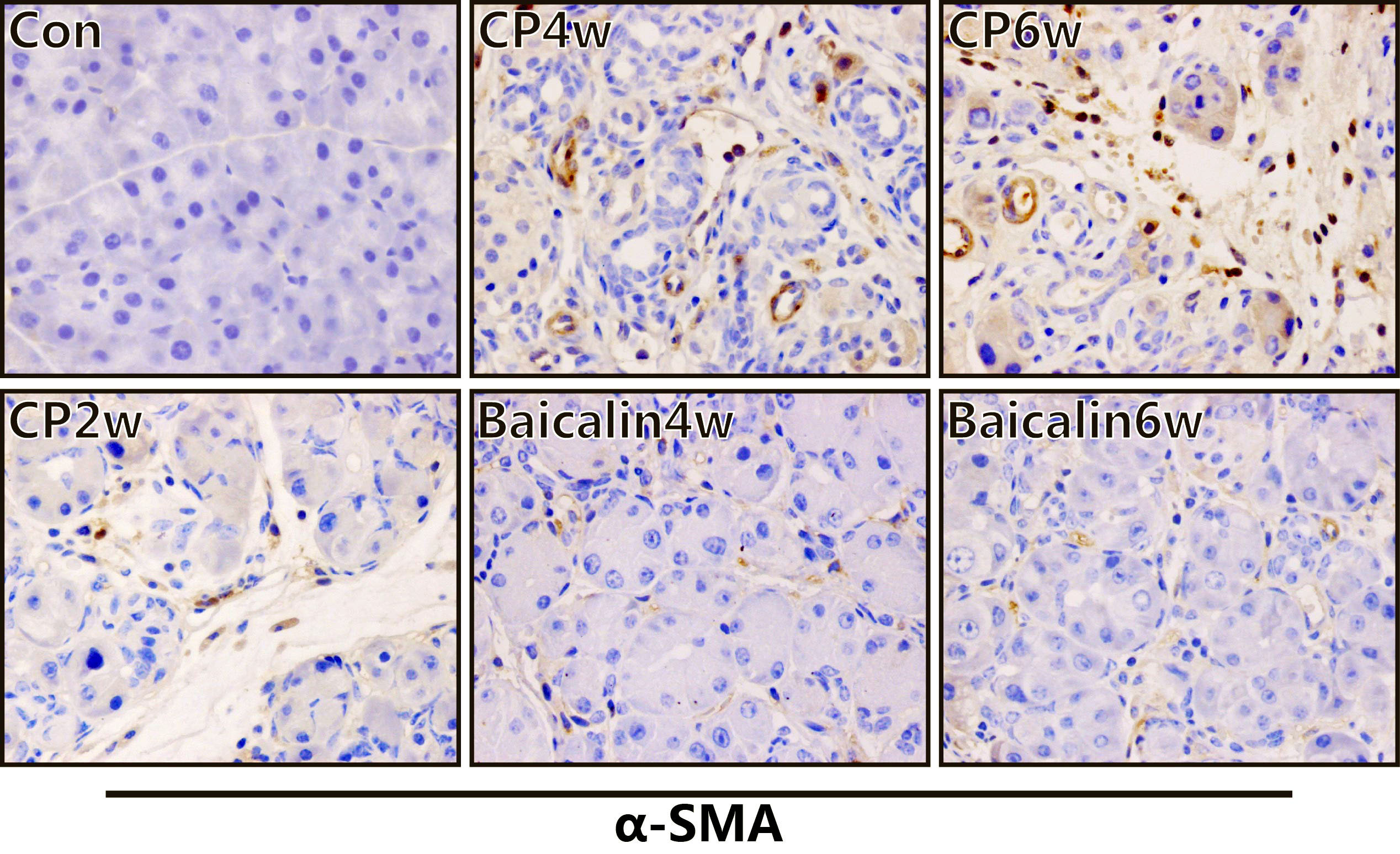

Supplement: Supplementary file 2 [file datasheet2.zip › figures-revised/figure 3/figure 3 (A).jpg]

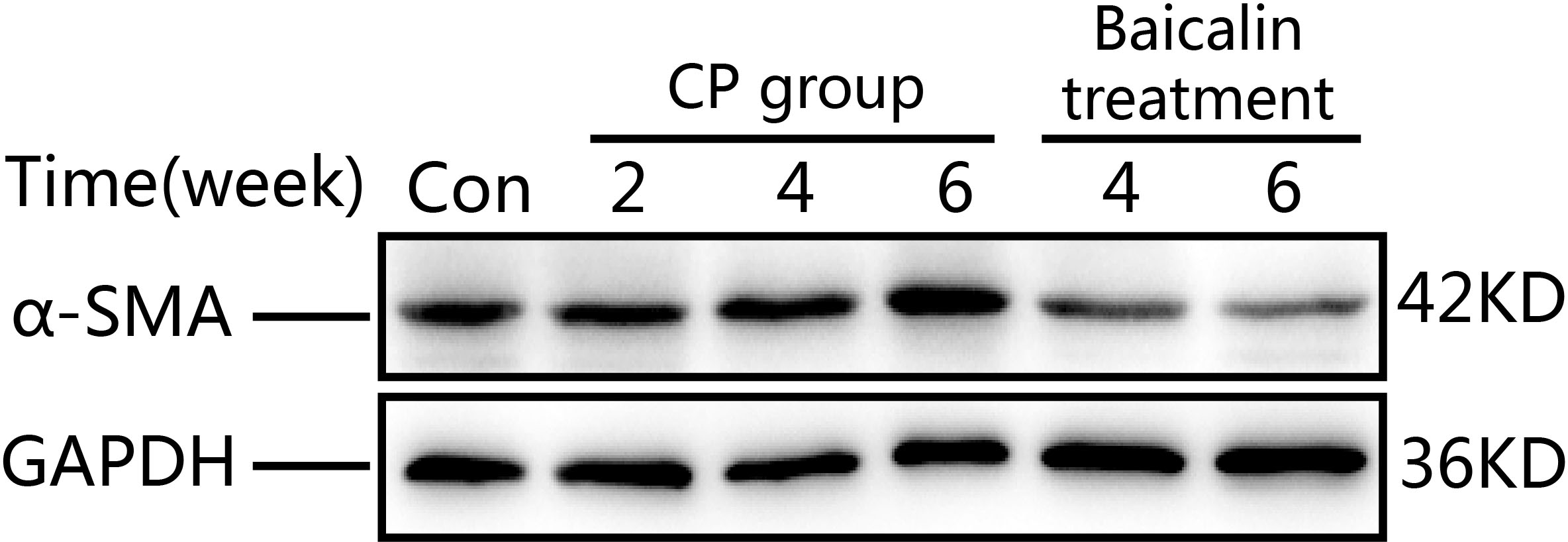

Supplement: Supplementary file 2 [file datasheet2.zip › figures-revised/figure 3/figure 3 (B)-1.jpg]

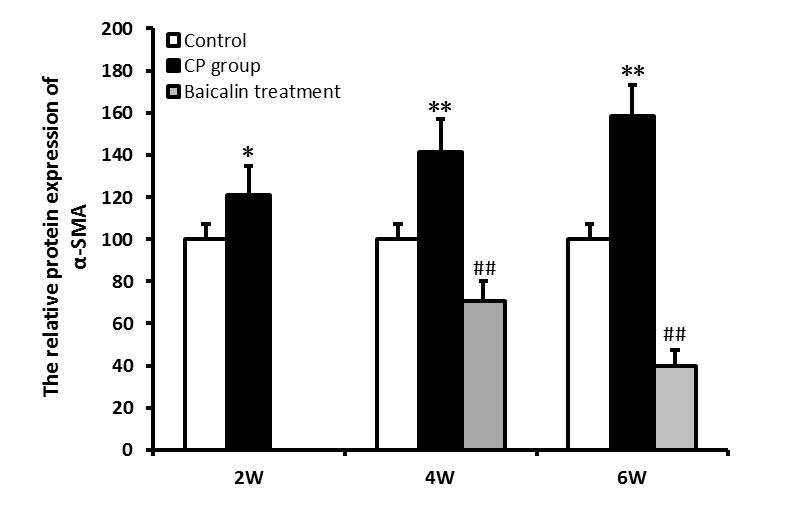

Supplement: Supplementary file 2 [file datasheet2.zip › figures-revised/figure 3/figure 3 (B)-2.jpg]

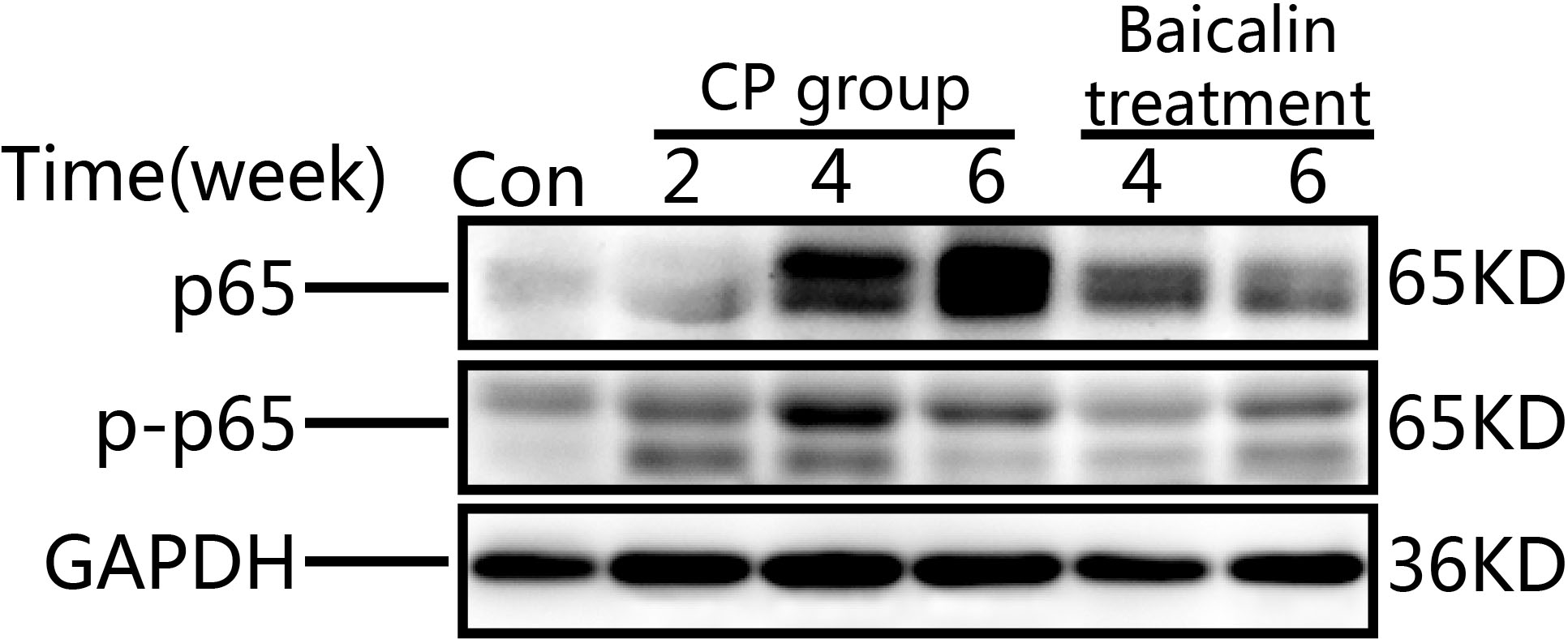

Supplement: Supplementary file 2 [file datasheet2.zip › figures-revised/figure 4/figure 4 (A)-1.jpg]

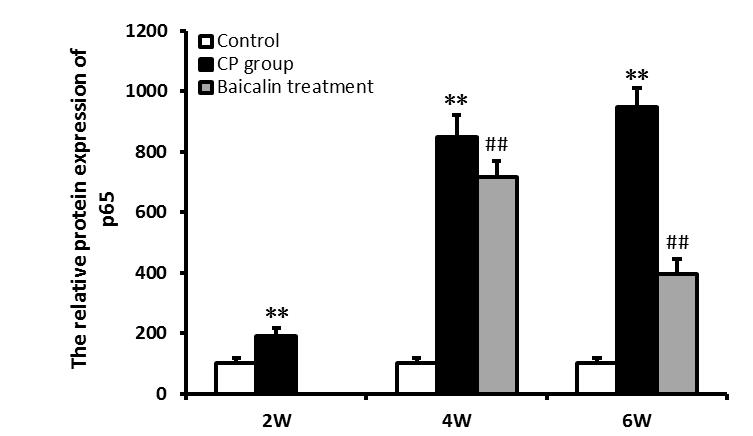

Supplement: Supplementary file 2 [file datasheet2.zip › figures-revised/figure 4/figure 4 (A)-2.jpg]

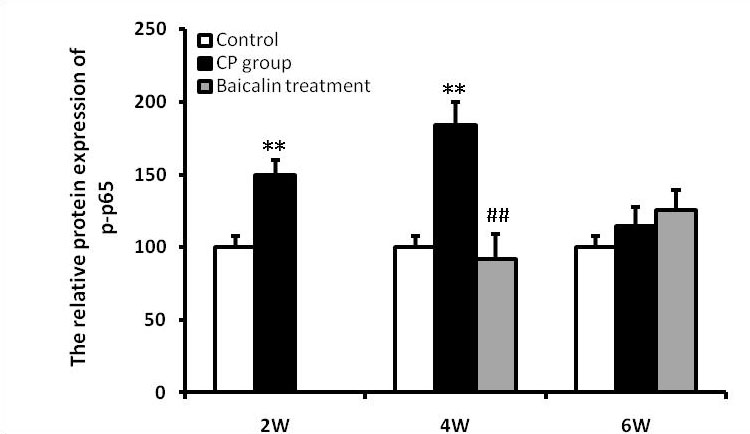

Supplement: Supplementary file 2 [file datasheet2.zip › figures-revised/figure 4/figure 4 (A)-3.jpg]

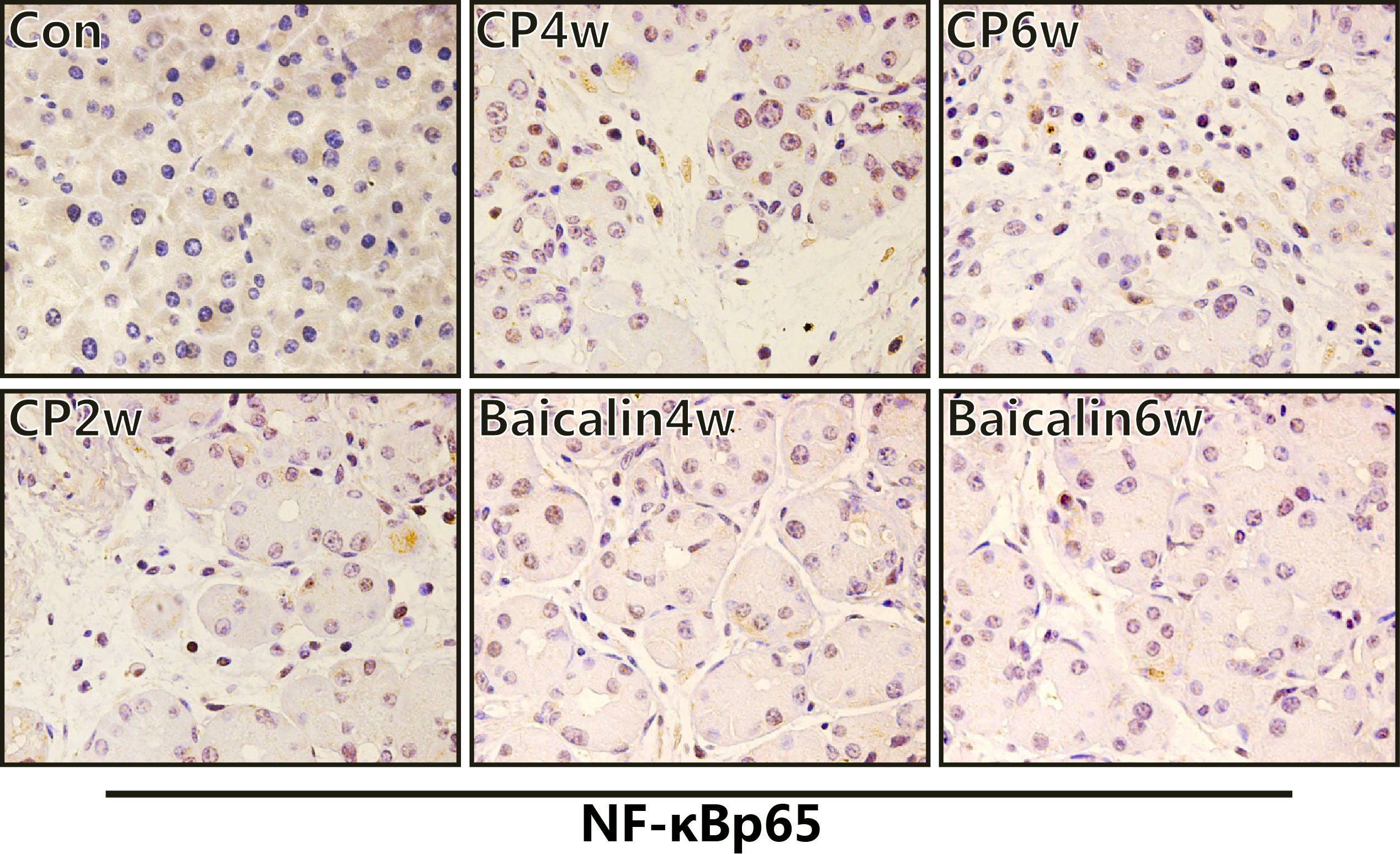

Supplement: Supplementary file 2 [file datasheet2.zip › figures-revised/figure 4/figure 4 (B).jpg]

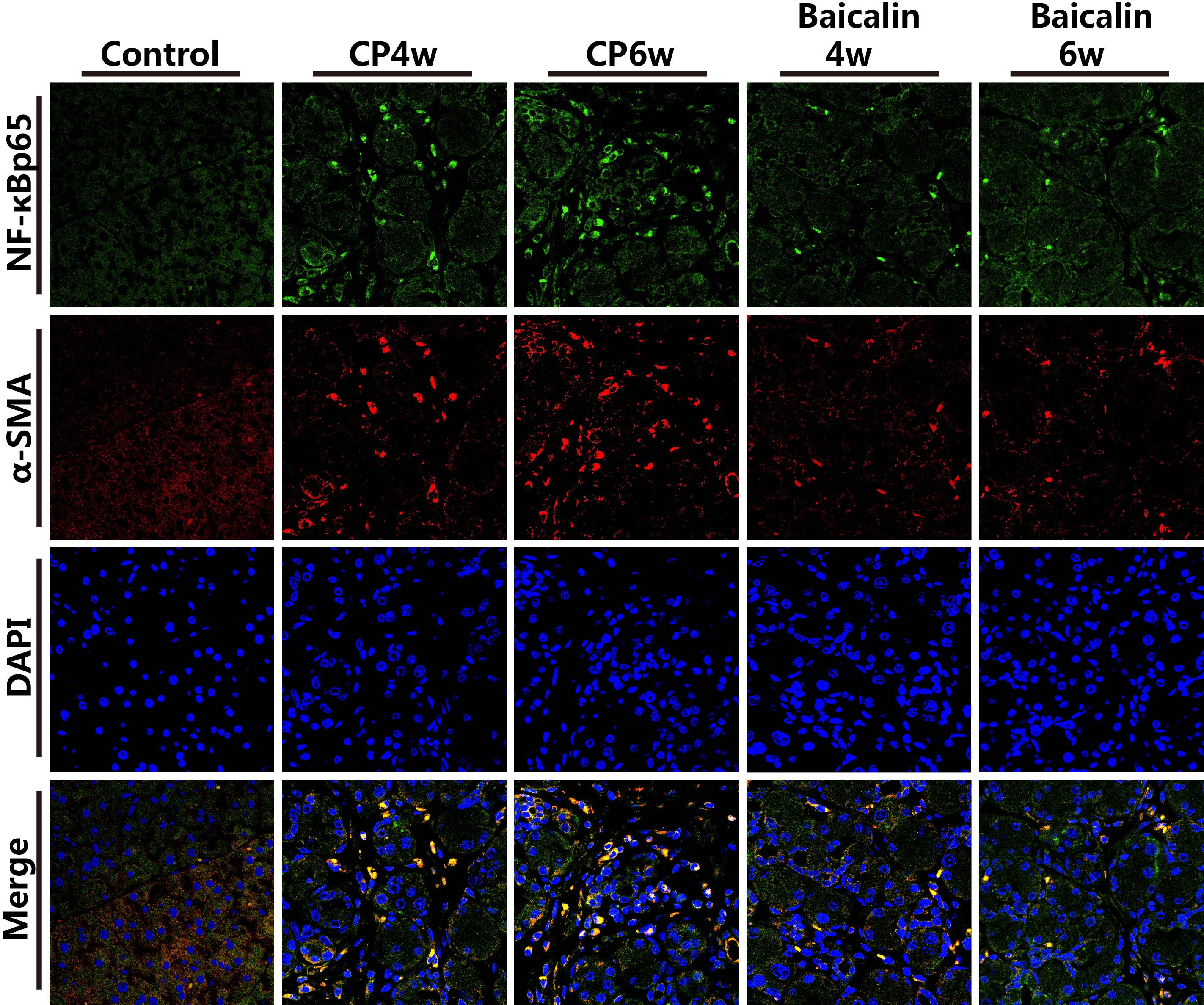

Supplement: Supplementary file 2 [file datasheet2.zip › figures-revised/figure 4/figure 4 (C).jpg]

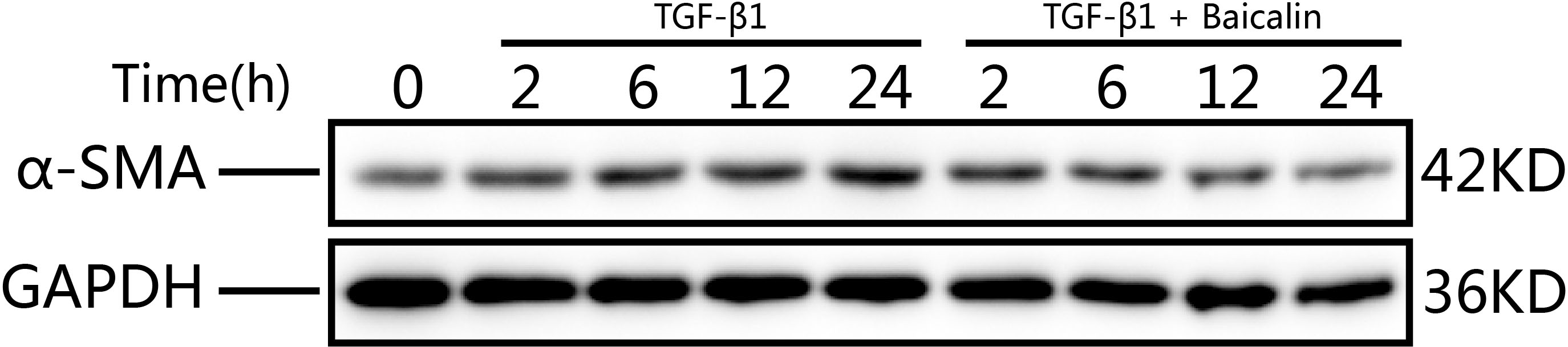

Supplement: Supplementary file 2 [file datasheet2.zip › figures-revised/figure 4/figure 4 (D)-1.jpg]

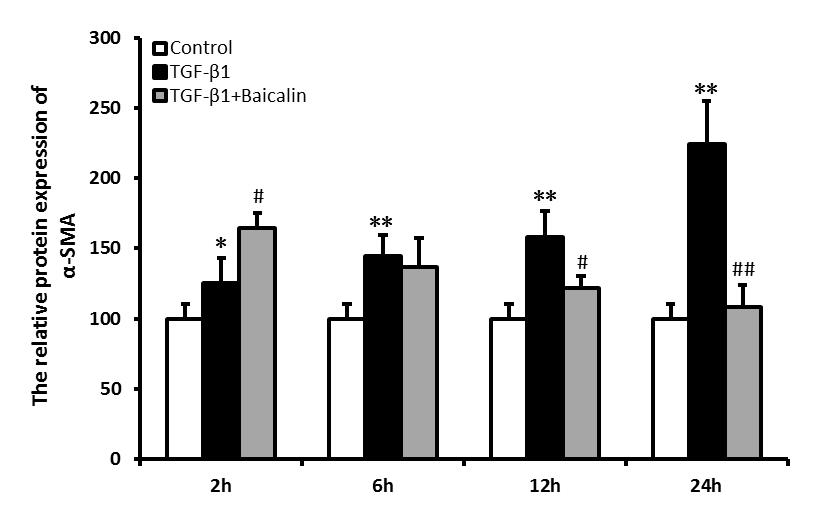

Supplement: Supplementary file 2 [file datasheet2.zip › figures-revised/figure 4/figure 4 (D)-2.jpg]

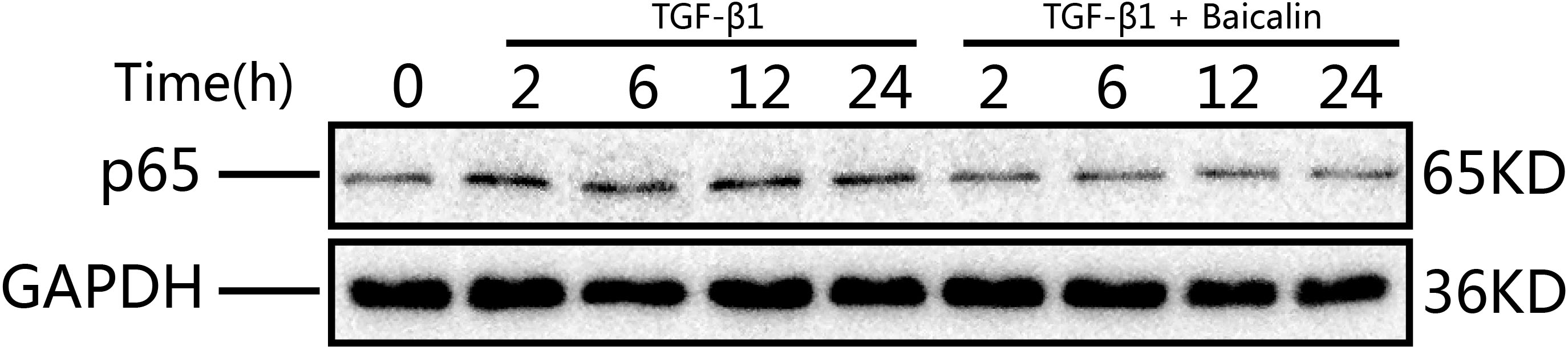

Supplement: Supplementary file 2 [file datasheet2.zip › figures-revised/figure 4/figure 4 (E)-1.jpg]

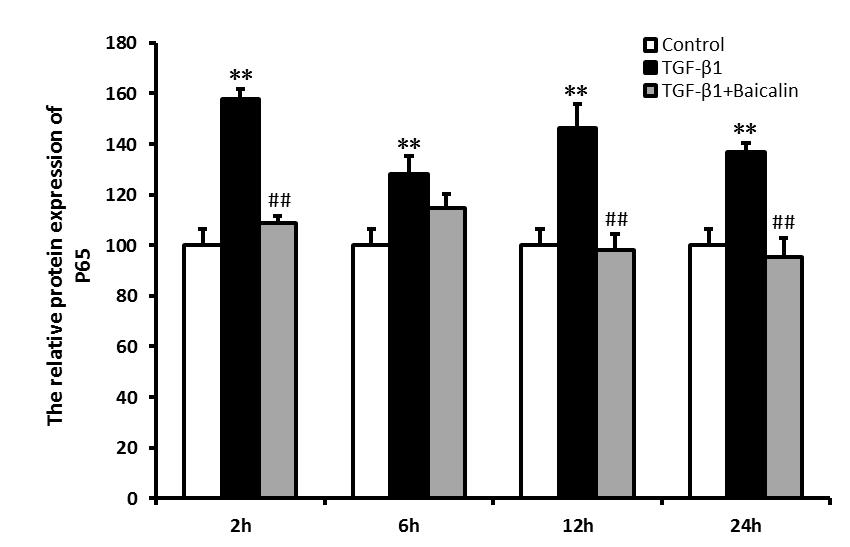

Supplement: Supplementary file 2 [file datasheet2.zip › figures-revised/figure 4/figure 4 (E)-2.jpg]

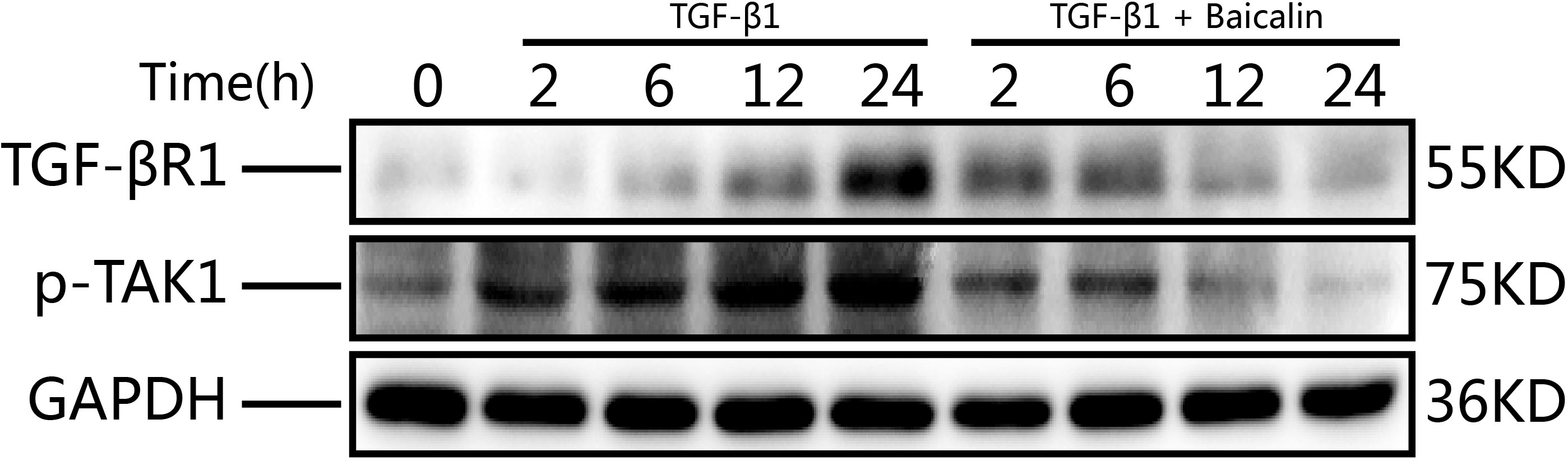

Supplement: Supplementary file 2 [file datasheet2.zip › figures-revised/figure 4/figure 4 (F)-1.jpg]

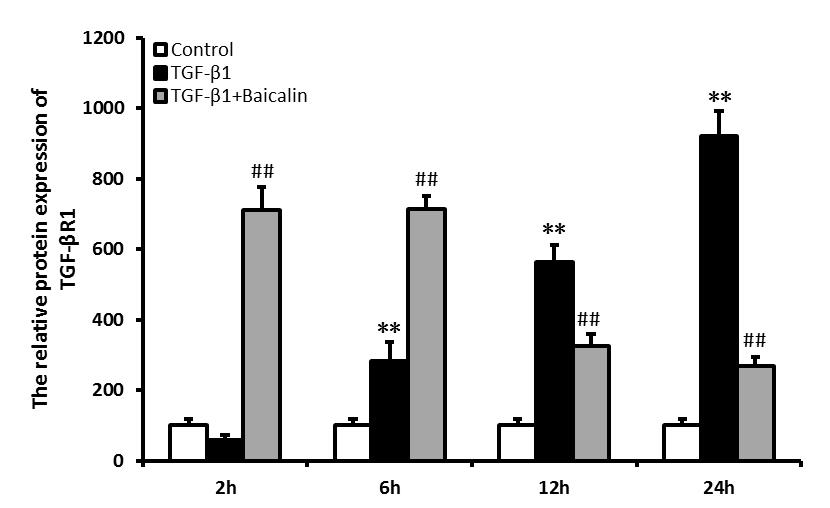

Supplement: Supplementary file 2 [file datasheet2.zip › figures-revised/figure 4/figure 4 (F)-2.jpg]

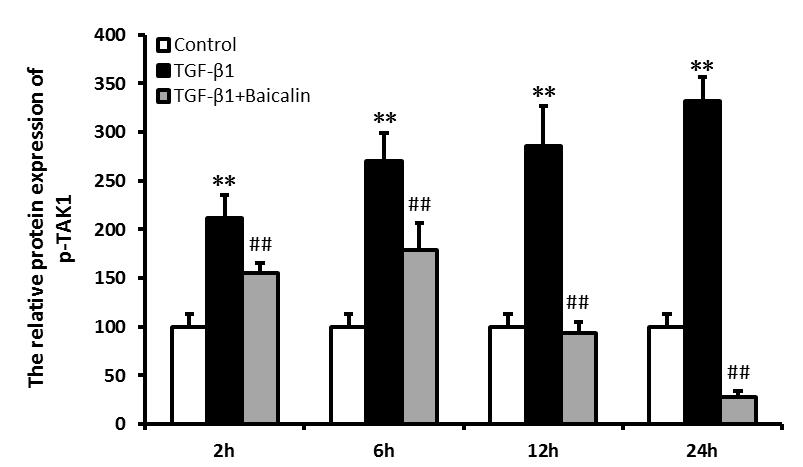

Supplement: Supplementary file 2 [file datasheet2.zip › figures-revised/figure 4/figure 4 (F)-3.jpg]

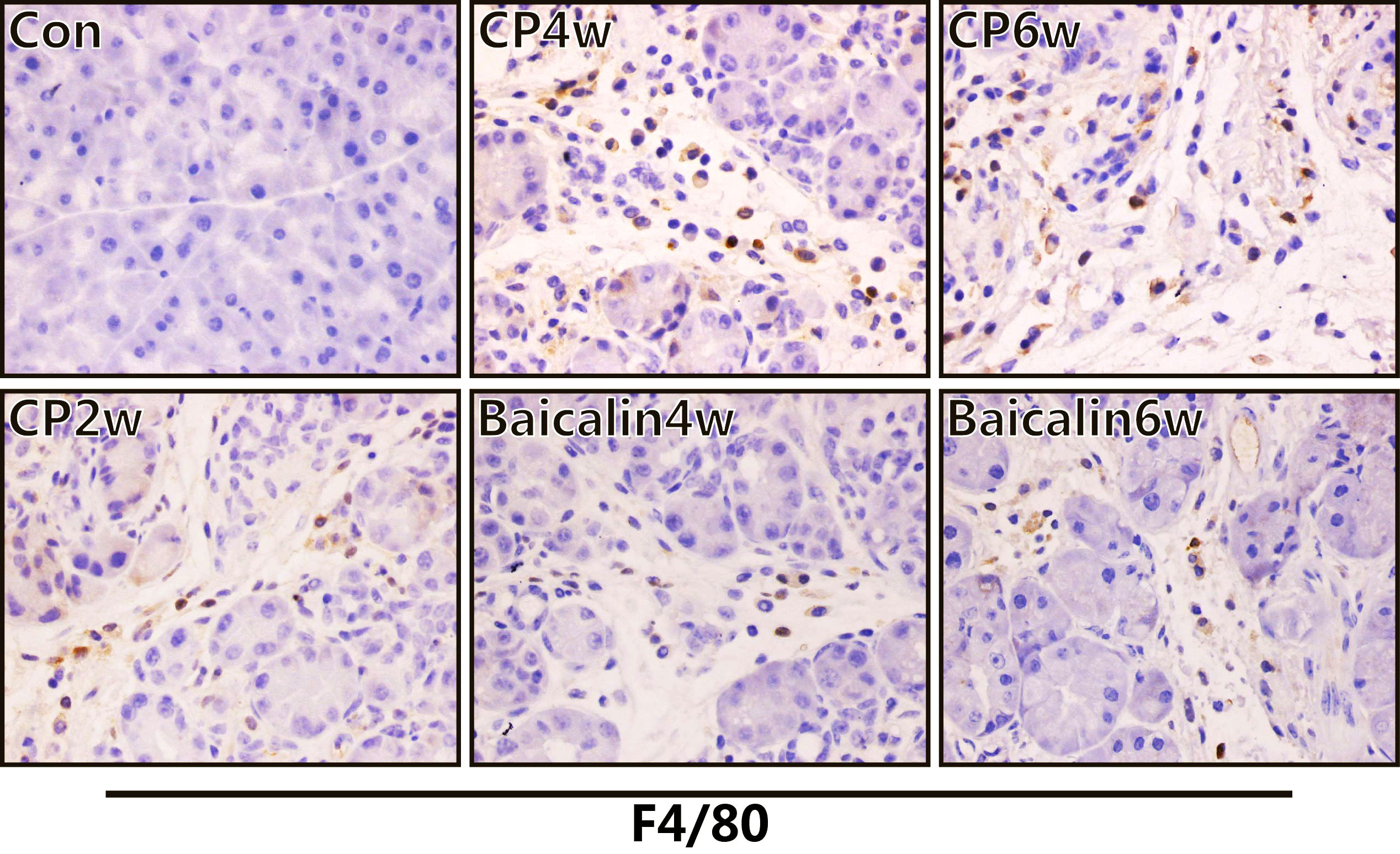

Supplement: Supplementary file 2 [file datasheet2.zip › figures-revised/figure 5/figure 5 (A).jpg]

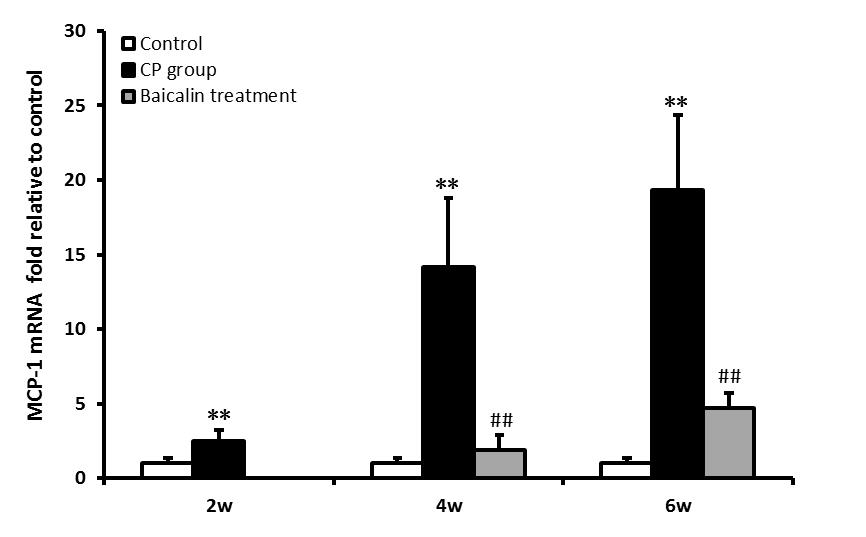

Supplement: Supplementary file 2 [file datasheet2.zip › figures-revised/figure 5/figure 5 (B).jpg]

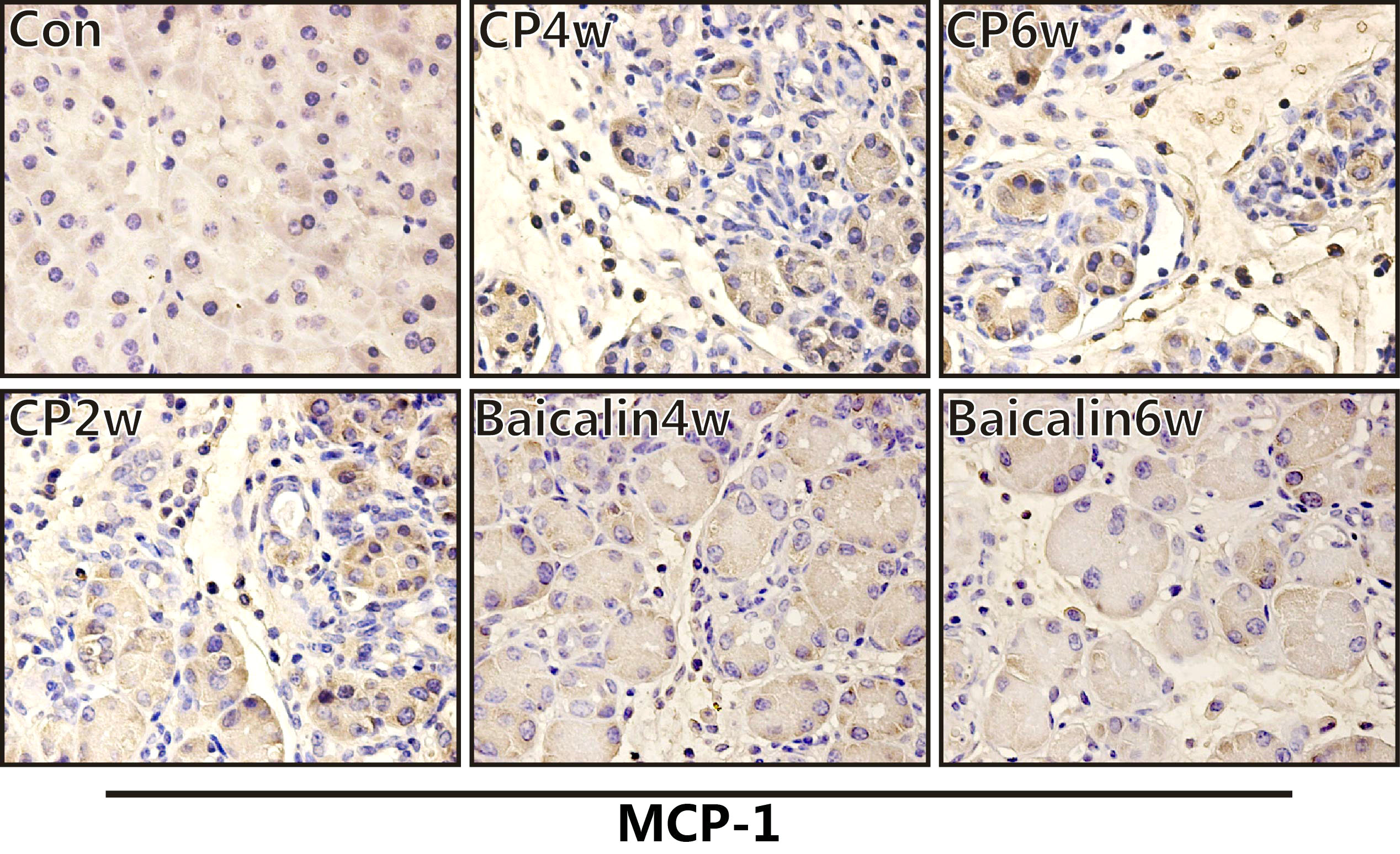

Supplement: Supplementary file 2 [file datasheet2.zip › figures-revised/figure 5/figure 5 (C).jpg]

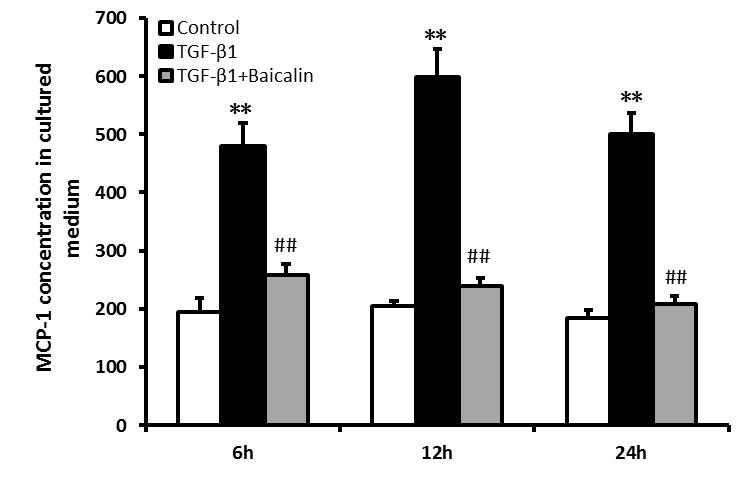

Supplement: Supplementary file 2 [file datasheet2.zip › figures-revised/figure 5/figure 5 (D).jpg]

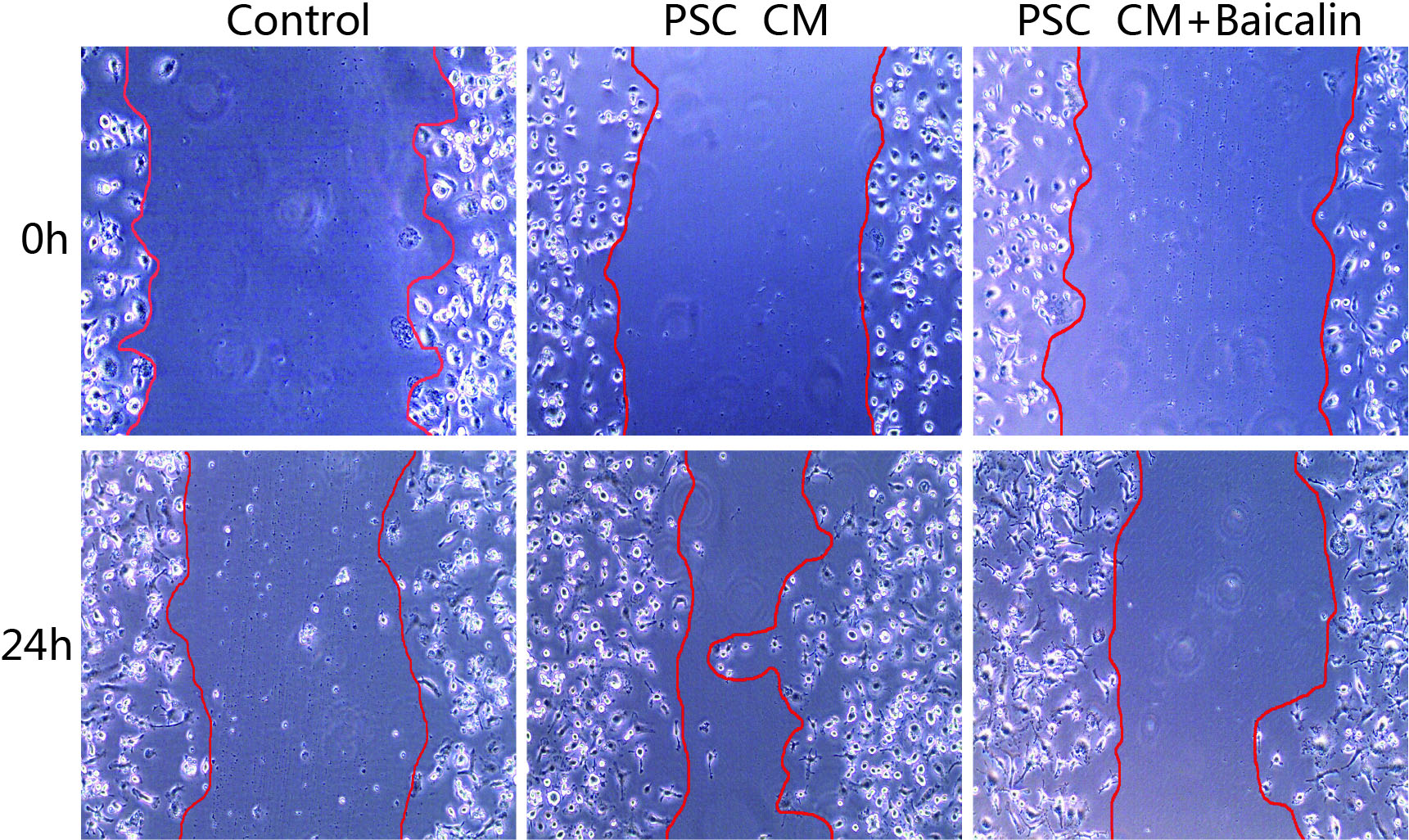

Supplement: Supplementary file 2 [file datasheet2.zip › figures-revised/figure 5/figure 5 (E)-1.jpg]

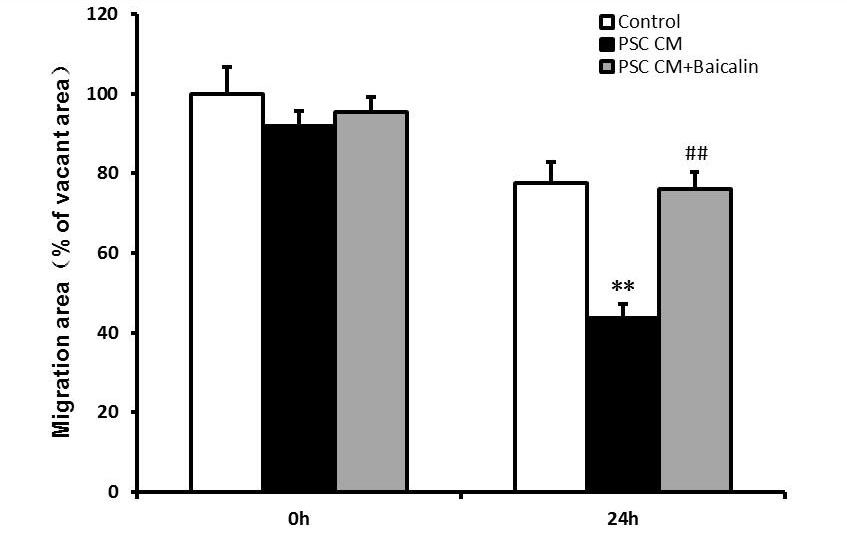

Supplement: Supplementary file 2 [file datasheet2.zip › figures-revised/figure 5/figure 5 (E)-2.jpg]

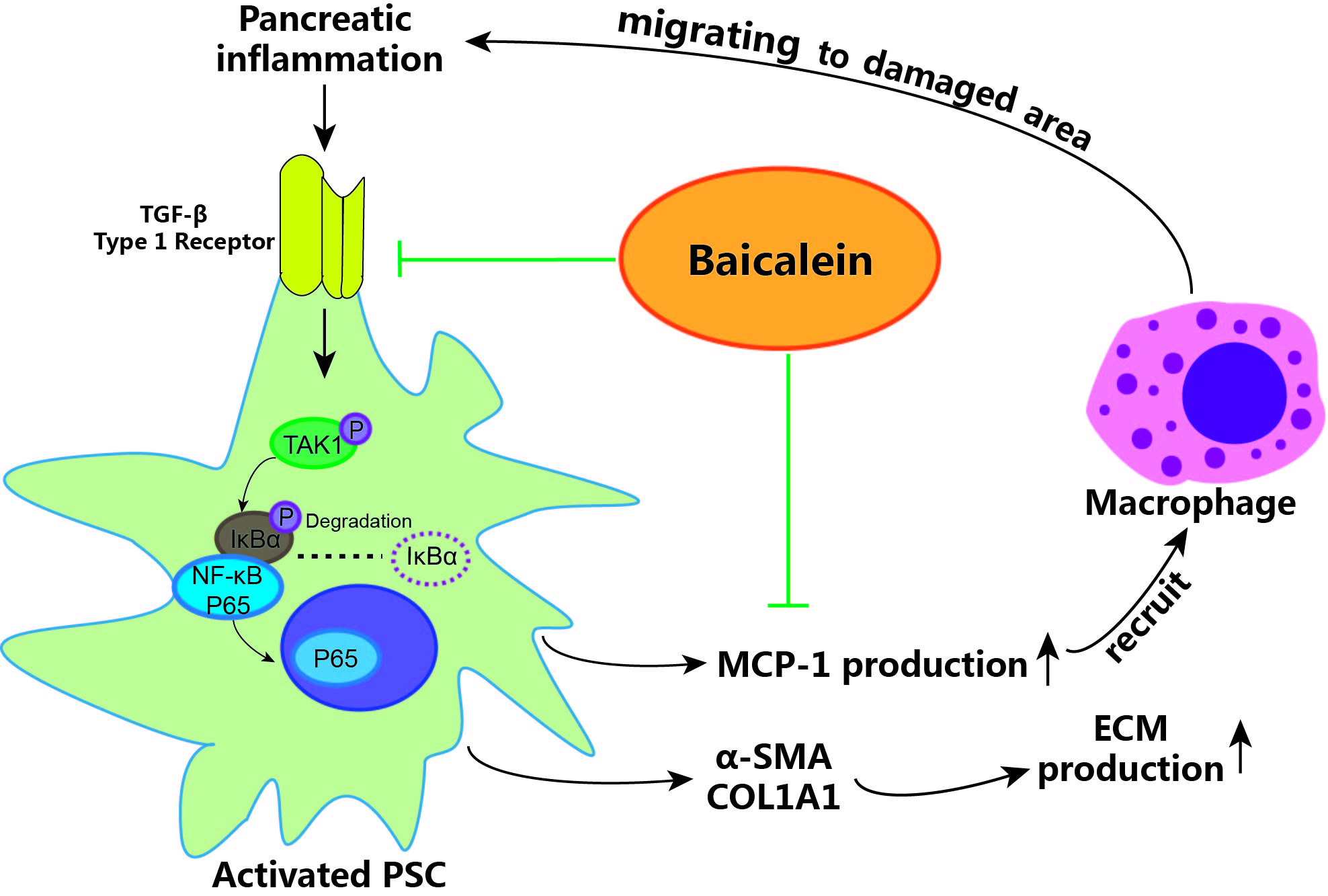

Supplement: Supplementary file 2 [file datasheet2.zip › figures-revised/Figure 6/Figure6.jpg]
